# Inactive Tlk associating with Tak1 increases p38 MAPK activity to prolong the G2 phase

**DOI:** 10.1038/s41598-018-36137-1

**Published:** 2019-02-13

**Authors:** Gwo-Jen Liaw, Chuen-Sheue Chiang

**Affiliations:** 1 Department of Life Sciences and Institute of Genome Sciences, National Yang-Ming University, Taipei, 112-22 Taiwan Republic of China; 2 Center for Diagnostics and Vaccine Development, Centers for Disease Control, Taipei, 115-61 Taiwan Republic of China; 3 School of Nursing, National Taipei University of Nursing and Health Sciences, Taipei, 112-19 Taiwan Republic of China

## Abstract

To guard genome integrity, response mechanisms coordinately execute the G2/M checkpoint in responding to stress. p38 MAPK is activated to prolong the G2 phase for completion of damage repair. Tlk activity is required for DNA repair, chromosome segregation and G2 recovery. However, the involvement of Tlk in G2 recovery differs from previous findings that *Tlk* overexpression delays the G2/M transition. To clarify this difference, genetic interaction experiments were performed using the second mitotic wave as model system. The results indicate that *Tlk* overexpression prolongs the G2 phase through *p38 MAPK* activation, independent of Tlk kinase activity. The results of co-immunoprecipitation, database search and RNAi screening suggest that *eEF1α1* and *Hsc70-5* links *Tlk* to *Tak1*. Reduced gene activities of *Tlk*, *Hsc70-5*, *eEF1α1* and/or *Tak1* couldn’t prolong the G2 phase induced by heat shock, indicating that these proteins work together to elevate *p38 MAPK* activity. In contrast, a high level of wild type Tlk decreases phosphorylated p38 MAPK levels. Thus, the difference is explained by a dual function of *Tlk*. When under stress, inactive Tlk increases p38 MAPK activity to prolong the G2 phase, and then activated Tlk modulates activities of p38 MAPK and Asf1 to promote G2 recovery afterwards.

## Introduction

Accumulated information indicates that genome instability is related to age-related diseases, such as neurodegeneration, metabolic disorders and carcinogenesis, etc^1^. Genome instability can be caused by lesions on chromosomal DNA, called as DNA damage. Exogenous factors to induce the DNA damage are environmental stresses such as heat and genotoxic agents such as ultraviolet light or chemical mutagens. To counteract stress, sensing and response mechanisms in cell cycle checkpoints coordinate their functions to tackle the DNA damage in order to maintain the genome integrity in eukaryotic cells. Among several response mechanisms, p38 MAPK (abbreviated as p38) is activated to inhibit activity of Stg, a Drosophila Cdc25 phosphatase homolog, leading to the G2 arrest^2^. For promoting the G2 recovery, Tlk is an important regulator that phosphorylates Asf1 and/or Rad9 required for nucleosome assembly and DNA repair, respectively^3–5^.

The cell cycle is divided into G0/G1, S, G2 and M phases, and cytokinesis. Transition from G2 to M phase, known as G2/M transition, begins after the Cdk1/CycB and Cdk1/CycA protein complexes have been accumulated to a threshold level^6^. In G2 phase, Cdk1 is phosphorylated by Wee1 kinase and kept in the inactive status. Activated Stg removes the inhibitory phosphate groups on Cdk1. The cell then enters M phase^7^.

The MAPK family contains three major groups: p38, JNK and ERK. p38 preferentially responds to environmental stress, such as heat shock, ionizing radiation and bacterial infection. The activation of p38 is through phosphorylation on Thr and Tyr residues in the activation loop by regulatory kinases. The activated p38 facilitates tolerance of organisms to various stresses to increase survival of organisms^8^. In mammals, there are four isoforms of p38, *α*, β, γ and δ. They are differentially expressed and activated in tissues^9^. Among the isoforms, p38*α* is well characterized. Mutations in the *p38α* gene reduce cell survival under stress^8^. The *Drosophila* genome encodes three p38 isoforms, p38a, -b and -c. Only p38a and p38b are believed to be bona fide p38 proteins. The function of these two isoforms overlaps because fly homozygous for both mutants is lethal while fly homozygous for either *p38a* or *p38b* is viable^10^. Results from several studies have demonstrated their roles in the stress response^10,11^.

Tlk is a Ser/Thr protein kinase and is highly conserved among protozoans, plants and animals^12–14^. Mutations in the *Arabidopsis tsl* gene cause random loss of floral organs, implicating its function in cell division^15^. Two mammalian *tsl* homologues, *Tlk*1 and *Tlk2*, are identified and their kinase activities are maximally activated by DNA damage during the S phase^16,17^. These two kinases also participate in chromosomal segregation^17^. In *D*. *melanogaster* and *Caenorhabditis elegans*, a single gene encodes Tlk homologue. Tlk acts as a cofactor of Aur-B, which is required for spindle formation, chromosomal segregation and cytokinesis^13,18,19^. Chromosomal missegregation is observed in embryos lacking *Tlk* activity^12,18^, indicating that *Tlk* activity is required for maintaining genome integrity.

As mentioned above, both *Tlk*1 and *Tlk2* activities promote the G2 recovery from the G2 arrest induced by DNA damage^3,5^. This differs from the findings of ours and others’ that overexpression of *Tlk* or *TLK2* impairs the G2/M transition^20,21^. To clarify the role of Tlk in the G2/M transition, we mainly performed genetic interaction experiments using the second mitotic wave (SMW) in *Drosophila* eye disc as a model system^22^.

## Results

### *Tlk* overexpression prolongs the G2 phase

**Figure 1:**
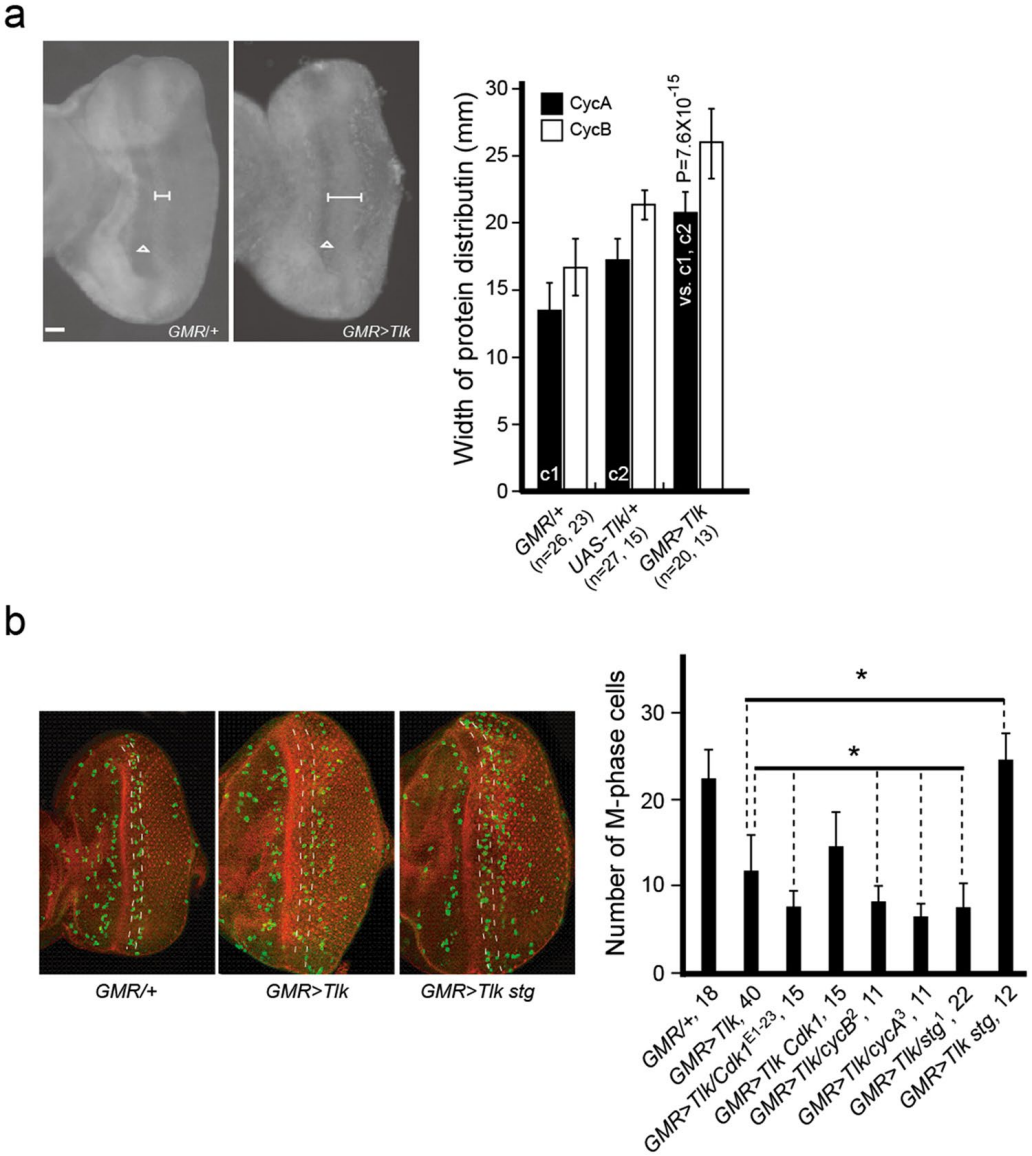
*Tlk* overexpression delays the G2/M transition. (**a**) *Tlk* overexpression results in a widened distribution of CycA. Eye-antennal discs were dissected from late third instar larvae immunostained with anti-CycA or anti-CycB antibody and photographed under fluorescence microscope. From hereafter, a detailed description for abbreviated genotypes is in Supplementary Table S1. Horizontal error bars in the photos delimit the distribution of CycA behind the morphogenetic furrow (MF) indicated by arrow heads. Scale bar is 10 μm. The bar graph shows the width of CycA (solid bars) and CycB (open bars) distribution behind MF (mean ± s.d.). Abbreviated genotypes are shown, followed by two numbers in parenthesis (see Immunostaining in Methods). For example, “*GMR*/+ (n = 26, 23)” indicates that 26 and 23 eye discs were used for measurement of the width of CycA and CycB distribution, respectively. The biological replicate was 26 and 23, respectively. To show the result of one way ANOVA test clearly, controls and test are marked in the bar graph. The two controls are marked as c1 and c2 whereas the test is marked as “vs. c1, c2”, indicating the test compared with the two controls c1 and c2. The P value is shown above the test bar. (**b**) Reduced *cdk1*, *cycA*, *cycB* or *stg* activity enhances the G2 delay. Reduced *CycB* activity means that the *CycB* activity remains ≥50% in eye disc heterozygous for *CycB*^2^ mutant (the end of Supplementary Table S1). The second mitotic wave (SMW) in eye disc was used to analyze function of the genes in the G2/M transition. The immunostaining with anti-phosphohistone H3 antibody was used to reveal and show M phase cells (green) in eye disc of *GMR*/+, *GMR* > *Tlk* and *GMR* > *Tlk stg*. Phalloidin-TRIC was used to stain contour of the neuronal cluster (red). The two dashed yellow lines delimit area of the 4–6 rows of neuronal clusters. The posterior of the eye disc is arranged toward the right. The bar graph shows the number of M-phase cell in the 4–6 rows of neuronal clusters counted under confocal microscope (mean ± s.d.). Prolonged G2 phase leads to few M phase cells in this regions. Abbreviated genotypes of the eye disc are shown, followed by the number of eye disc (biological replicate) measured (see Immunostaining in Methods). The asterisks indicate that the number of M-phase cell in *GMR* > *Tlk*^T2^ eye discs with either co-overexpression or reduction of various genes was significantly different from that in *GMR* > *Tlk*^T2^ discs (two tailed analysis of Student’s *t* test; *P < 0.05).

Our previous study shows that overexpression of wild type *Tlk* in *UAS*-*Tlk*^T2^ transgene driven by *GMR*-*GAL4* causes a shift of where most cells complete their cell division from the 4–6 rows of neuronal clusters to the 7–10^21^. Complete genotypic information is in Supplementary Table S1, including the *UAS* transgenic flies. This delayed cell division may be a result of prolonged either S phase or/and G2 phase. In eye disc behind the morphogenetic furrow (MF) with *Tlk* overexpression, the S phase progression was not affected as seen by the bromodeoxyuridine labeling (Supplementary Fig. S1), while the G2 phase was prolonged as evidenced by the significantly widened distribution of CycA by immunostaining (Fig. 1a). Results from the genetic interaction studies further show that the prolonged G2 phase, leading to few M phase cells in the 4–6 rows of neuronal clusters (defined in Methods), was enhanced by reduced either *cycA* or *cycB* activity (Fig. 1b). Similarly, the G2 delay was also enhanced by reduced either *Cdk*1 or *stg* activity and suppressed by *stg* overexpression (Fig. 1b). In summary, *Tlk* overexpression causes the G2 delay.

### The overexpressed *Tlk* acts through *p38* to prolong the G2 phase

**Figure 2:**
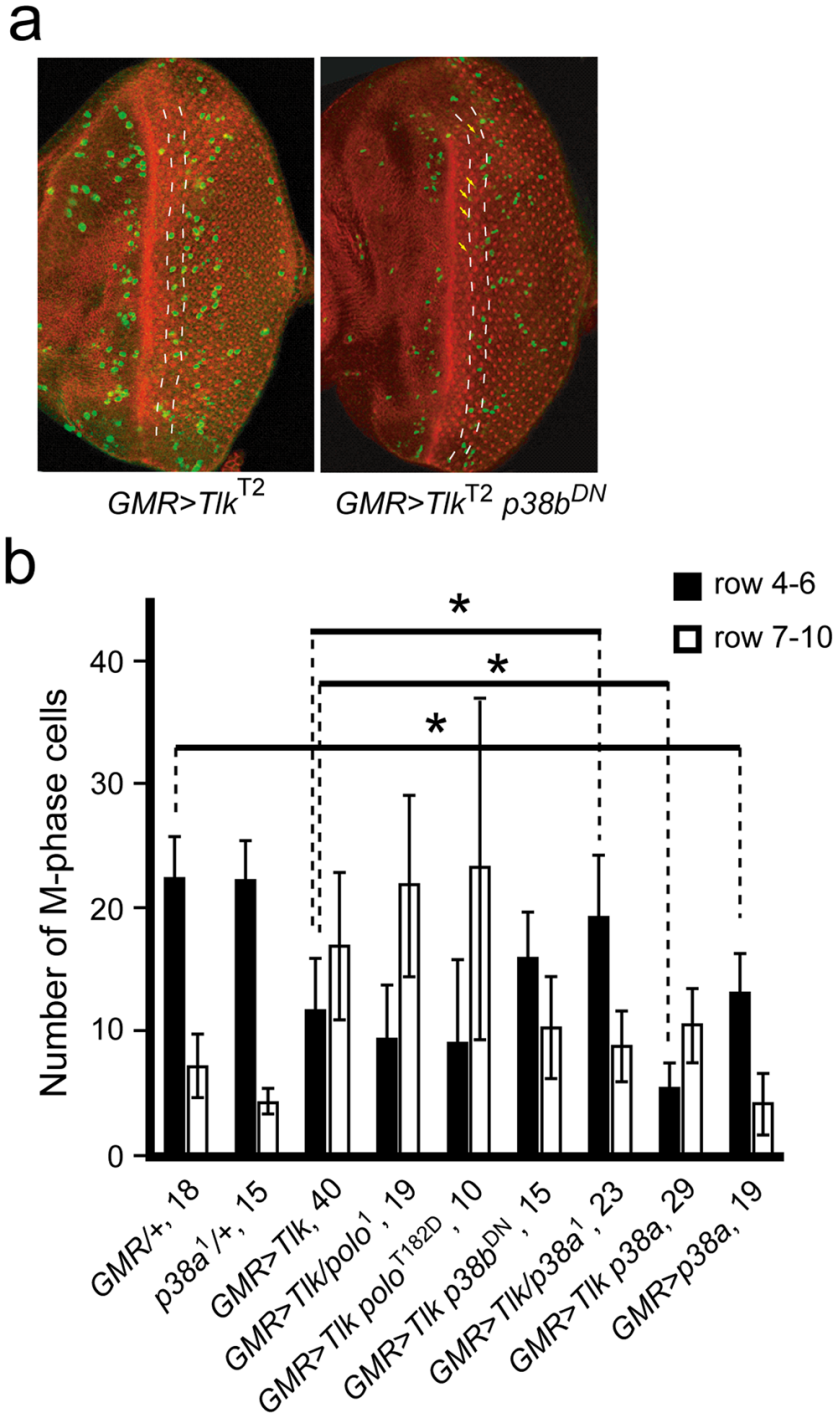
Reduced *p38a* activity suppresses the prolonged G2 phase. (**a**) Immunostaining with anti-phosphohistone H3 antibody (green) was used to determine the number of M phase cell in the 4–6 rows of neuronal clusters of eye disc with *Tlk* overexpression (*GMR* > *Tlk*^T2^), and with co-overexpression of *Tlk* and a dominant negative *p38b* (*GMR* > *Tlk*^T2^ *p38b*^DN^). Phalloidin-TRIC was used to stain the neuronal clusters (red). Two dashed yellow lines delimit area of the 4–6 rows of neuronal clusters. The posterior of the eye disc is arranged toward the right. *Tlk* and *p38b*^DN^ co-overexpression results in disordered positioning of nuclei in the eye disc, leading to some M phase cells on the same focal plane with smaller and weaker signals, indicated by yellow arrows. This phenotype is also observed in follicle cells with *Tlk* loss-of-function^32^. (**b**) The bar graph shows the number of M phase cell in the 4–6 rows of neuronal clusters. Abbreviated genotypes of the eye discs are shown, followed by the number of eye disc **(**biological replicate) measured under confocal microscope (mean ± s.d.) (see Immunostaining in Methods). *GMR* > *Tlk*^T2^/*polo*^1^ and *GMR* > *Tlk*^T2^/*p38a*^1^ represent *Tlk* overexpression in eye disc heterozygous for either *polo*^1^ or *p38a*^1^mutant, respectively. The asterisks indicate that the number of M phase cell in the 4–6 rows of neuronal clusters in *GMR* > *Tlk*^T2^ discs with either reduction or co-overexpression of various genes was significantly different from that in *GMR* > *Tlk*^*T*2^ discs (two tailed analysis of Student’s test; *P < 0.05).

Besides activated p38, activation of Polo kinase or microtubule catastrophe also induces the G2 arrest. Activated Polo kinase positively and negatively regulates the Wee1 and Stg activities, respectively, to inhibit Cdk1 activity for progression of the G2/M transition^23^. Microtubule catastrophe activates protein phosphatase 2A (PP2A) to inhibit Stg activity, leading to the G2/M arrest^23^. In our study, the defect on microtubule morphology in eye disc with *Tlk* overexpression was subtle (Supplementary Fig. S2), indicating that microtubule catastrophe is unlikely. Therefore, the genetic relationship of *Tlk* with *p38a*, *p38b* or *polo* was investigated. The G2 delay was caused by the overexpressed Tlk, which is one of the gain-of-functions. We performed the epistatic analysis based on an assumption that *Tlk* acts upstream these genes. Therefore, a reduction of the investigated gene activity was expected to suppress the G2 delay resulted from *Tlk* overexpression. To reduce gene activity, eye disc heterozygous for one of the corresponding mutants was used. The results of the epistatic analysis indicated that reduction of *p38a* activity suppressed the G2 delay, while *p38a* overexpression enhanced the G2 delay (Fig. 2). This is consistent with the finding that activated p38*α* induces the G2 delay in humans^2^. The suppression of the G2 delay by overexpression of *p38b*^DN^, a dominant negative allele, was not statistically significant. Changes of *polo* activity did not suppress the G2 delay (Fig. 2). Taken together, these results supported that *Tlk* is epistatic to *p38a*.

**Figure 3:**
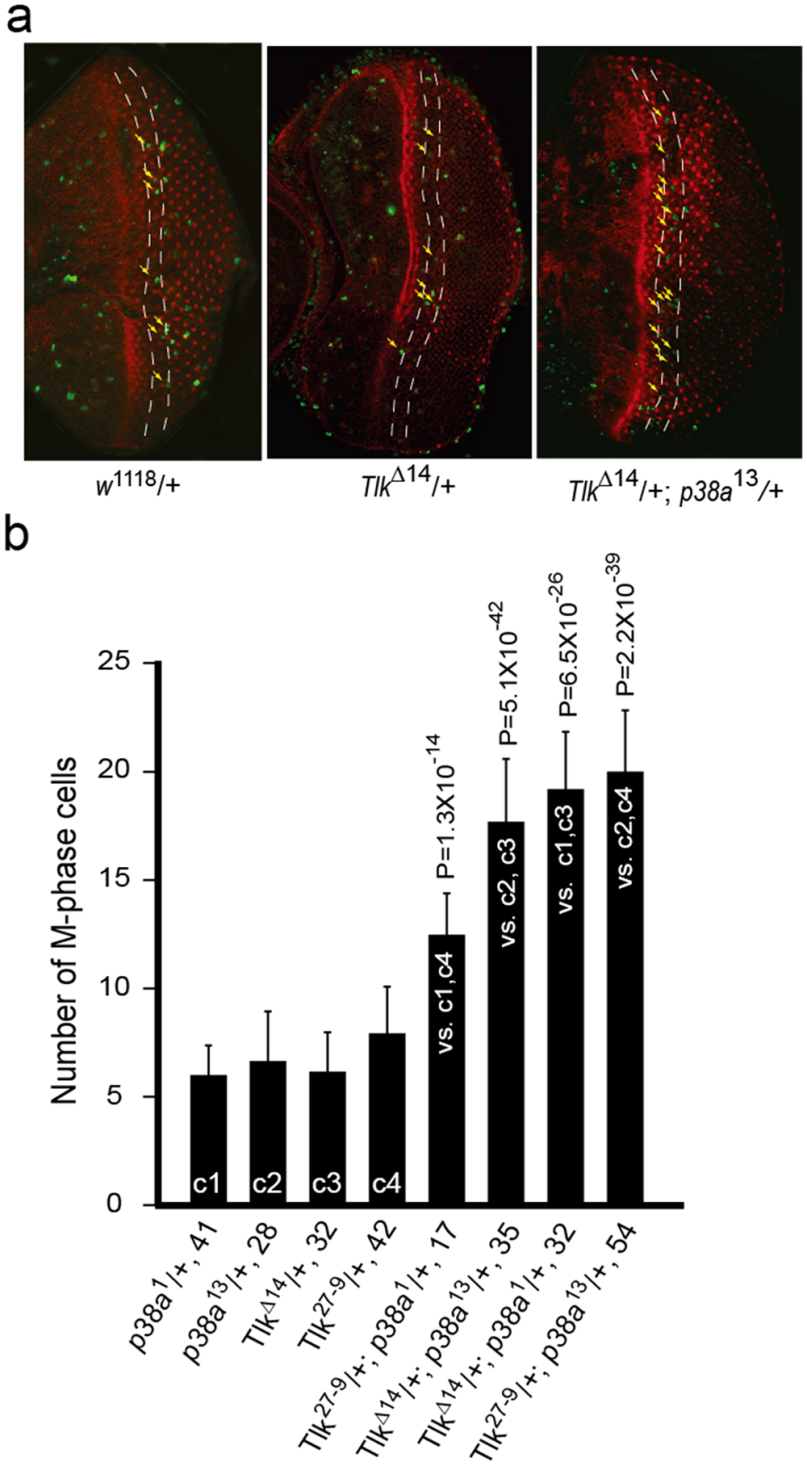
The G2 recovery occurs earlier in eye disc transheterozygous for both *Tlk* and *p38a* mutants. (**a**) Third instar larvae were heat shocked at 37 °C for 1 hour and aged at room temperature for 3 hours. Eye-antennal discs of *w*^1118^/+, *Tlk*^Δ14^/+ and *Tlk*^Δ14^/+; *p38a*^13^/+ were dissected to assess M phase cells by immunostaining with anti-phosphohistone H3 antibody (green). Phalloidin-TRIC was used to stain the neuronal cluster (red). The numbers of M phase cell in the 4–6 rows of neuronal clusters, delimited by two yellow dashed lines, were counted. Yellow arrows mark M phase cells with weaker signals, likely due to either at early M phase or the disordered positioning of nuclei. (**b**) The bar graph shows the number of M phase cell in the 4–6 rows of neuronal clusters. Abbreviated genotypes of the eye discs are shown, followed by the number of eye disc **(**biological replicate) measured (mean ± s.d.) (see Immunostaining in Methods). Controls and tests are marked in the bar graph. The controls, eye discs heterozygous for either *p38a* or *Tlk* mutant, are marked as c1, c2, c3 and c4. The tests, eye disc transheterozygous for both *p38a* and *Tlk* mutants, are marked as “vs. c1, c4”, “vs. c2, c3”, “vs. c1, c3” and “vs. c2, c4”. The statistical analysis using one way ANOVA test is described in Fig. 1a. The P value is shown above each test bar.

To further investigate *p38a*, the dosage dependent genetic interaction experiment was performed. The diagram at the end of Supplementary Table S1 illustrates this genetic concept. *Tlk*^Δ14^, *Tlk*^27–9^, *p38a*^1^ and *p38a*^13^ are recessive mutants. According to the definition of recessive mutation, no phenotype appears in eye disc heterozygous for one of the *Tlk* or *p38a* mutants. This manifests that the elevated *p38* activity is sufficient to prolong the G2 phase when under stress, although it is lower. The prolonged G2 phase results in less M phase cells in the 4–6 rows of neuronal clusters, as that in wild type disc. Thus, the numbers of M phase cell from wild type and from eye disc heterozygous for one of either *Tlk* or *p38a* mutants serve as controls in this experiment. In eye disc transheterozygous for *Tlk* and *p38a* mutants, served as tests (in the legend of Fig. 3b), both gene activities are reduced by ≤50%. If these two gene activities execute in the same pathway as indicated by the epistatic analysis, the remaining activity of the pathway is supposed to be 25%. This reduced activity would result in a lower p38 activity, therefore, the G2 recovery should occur earlier, leading to a significant number of M phase cell.

Activity of p38*α* is increased to prolong the G2 phase when cells are exposed to environmental stress. Once cellular damage is repaired, the level of activated p38*α* resumes to normal^8^. Heat shock was chosen as a stress for the dosage dependent genetic interaction experiment to increase p38 activity in eye disc, based on the facts that heat shock induces double-stranded DNA breaks^24^ and that kinase activity of Tlk is activated in responding to DNA damage^14,16^. Incubation at room temperature after heat shock allows the G2 recovery to occur.

Late third instar larvae were heat shocked at 37 °C for 1 hour and then incubated at room temperature for 3 hours. In the 4–6 rows of neuronal clusters, 4.2 ± 1.7 M phase cells were detected in the wild type disc, *w*^1118^/+, while 5.9 ± 1.7 to 7.8 ± 2.6 M phase cells were observed in control eye discs, which are heterozygous for either *Tlk* or *p38a* mutant (Fig. 3). The difference in the numbers of M phase cells was not significant among these eye discs. However, in eye disc transheterozygous for *Tlk* and *p38a* mutants, the number of M phase cell increased significantly, ranging from 12.4 ± 2.9 to 19.9 ± 3.8 (Fig. 3). This increase indicated a lower level of *p38* activity in eye disc transheterozygous for *Tlk* and *p38a* mutants and confirmed that *Tlk* is epistatic to *p38a*.

### Different Tlk levels differentially modulate P-p38 levels

Tlk is a Ser/Thr kinase and its kinase activity is activated when DNA damage occurs^4^. Thus, Tlk may increase p38 activity through protein phosphorylation. To overexpress *Tlk* ubiquitously, fly carrying a heat shock inducible *GAL4* transgene, *hs*-*GAL4*, was selected. In *hs* > *Tlk* embryos, Tlk levels were 1.3 to1.9 folds higher than that in the *hs*-*GAL4*/+ control embryo without stress (25 °C in Supplementary Fig. S3c). This slight increase may resemble the low level of Tlk driven by *GMR*-*GAL4* in MF. Therefore, cold shock was chosen to avoid further increase of Tlk level, based on the report that cold shock does not increase expression of the *hsp* genes^25^. The result was as expected that Tlk levels were similar in embryos with four different genotypes (12 °C in Supplementary Fig. S3c).

**Figure 4:**
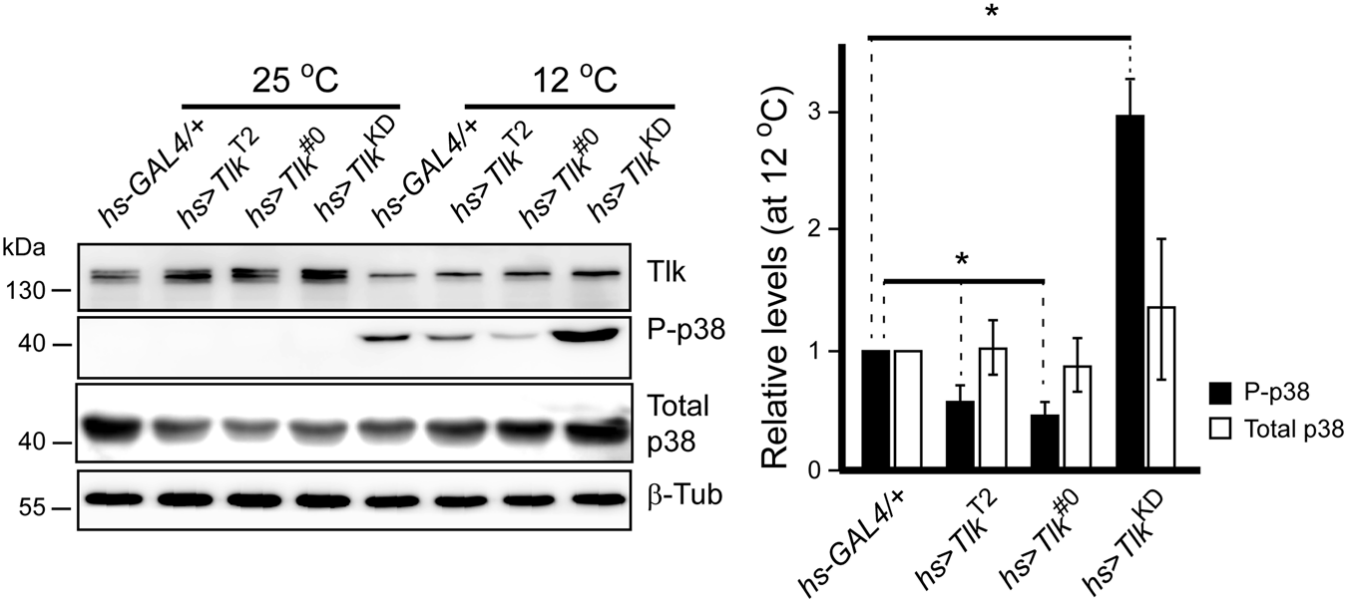
Wild type and inactive Tlk significantly decreases and increases P-p38 levels, respectively. Embryos of *hs*-*GAL4*/+, *hs* > *Tlk*^T2^, *hs* > *Tlk*^#0^ or *hs* > *Tlk*^KD^ were collected for 6 hours, aged for 12 hours and cold shocked at 12 °C for 25 min. KD stands for a kinase dead mutation. Controls were embryos without the cold shock (25 °C). Proteins were extracted from these treated and untreated embryos for detection of Tlk, phospho-p38 MAPK (P-p38), p38 and β-Tubulin (β-Tub) by western blotting. Four independent batches of embryonic extract were prepared (biological replicate) and two repeats were performed for each batch (technical replicate). Quantities of P-p38, total p38 and β-Tub were measured using Image-J. All values of P-p38 and total p38 were first normalized by the values of β-Tub and then by the value of *hs-GAL4*/+ to obtain relative levels of P-p38 (solid bars) and total p38 (open bars) (mean ± s.d.). The asterisks indicate that the relative levels of P-p38 in *hs* > *Tlk* embryo was significantly different from that in *hs*-*GAL4*/+ (two tailed analysis of Student’s *t* test; *P < 0.001).

Phosphorylation on Thr-180 and Ser-182 activates kinase activity of p38^26^. P-p38 represents the active form of p38. Therefore, the anti-P-p38 antibody was used to determine the P-p38 levels in *hs*-*GAL4*/+, *hs* > *Tlk*^T2^, and *hs* > *Tlk*^#0^ embryos by western blotting. The results showed that overexpression of wild type *Tlk* significantly decreased the P-p38 levels by approximately 50% in either *hs* > *Tlk*^T2^ or *hs* > *Tlk*^#0^ embryo (0.58 ± 0.16 and 0.48 ± 0.11 folds in Fig. 4). This is inconsistent with the conclusion that *Tlk* genetically increases the *p38a* activity. The decreased P-p38 level was also observed in *hs* > *Tlk*^#0^ adult with 15-minute heat shock at 38.5 °C followed by 45-minute incubation at room temperature (Supplementary Fig. S4). Unexpectedly, the P-p38 level was significantly, although marginally, higher in *UAS*-*Tlk*^#0^/+ adults (Supplementary Fig. S4), in which the Tlk level was lower, although insignificantly, than that in *hs* > *Tlk*^#0^ adults (Supplementary Fig. S4). The elevation might be due to more inactive form of Tlk when its level is low. We then tested whether inactive Tlk was able to increase P-p38 level using *UAS-Tlk*^KD^ transgenic fly that encodes an inactive form of Tlk^18^. After cold shock to *hs* > *Tlk*^KD^ embryo at 12 °C for 25 min, the P-p38 level was increased by 2.98 ± 0.29 folds, when compared to that in the *hs*-*GAL4*/+ embryo (Fig. 4). These results supported that inactive Tlk elevated P-p38 level. Furthermore, this elevation was independent of the levels of total p38. In summary, different Tlk levels differentially modulate P-p38 levels.

### Inactive Tlk works with Tak1 to increase p38 activity

To identify Tlk^KD^ associated proteins that would increase P-p38 level, a series of three screening experiments was designed. The first screening experiment was to find protein candidates that interact with Tlk from public databases, mainly from BioGRID. The second was to test whether the genes encoding these candidates genetically interact with *Tlk*, as described in Supplementary Fig. S5. The third was to perform the epistatic analysis to reveal its function in the G2 phase. The dosage dependent genetic interaction experiment was used as the final method to confirm its interaction with *Tlk*. The *UAS*-RNAi transgenic flies were used in the third screening experiments. Their off-target problem would be solved in the final experiments.

The first group of candidates was the genes encoding component proteins in the dSTRIPAK complex^27^ due to the data in BioGRID that shows a direct link of Tlk to dSTRIPAK through the two components, Fgop2 and SLMAP (Supplementary Fig. S5a). However, the results from the second screening experiments did not support that *Tlk* genetically interacts with *mts*, encoding the PP2A catalytic subunit of dSTRIPAK (Supplementary Fig. S5b). This is consistent with the elimination of PP2A described earlier regarding microtubule catastrophe (Supplementary Fig. S2).

**Figure 5:**
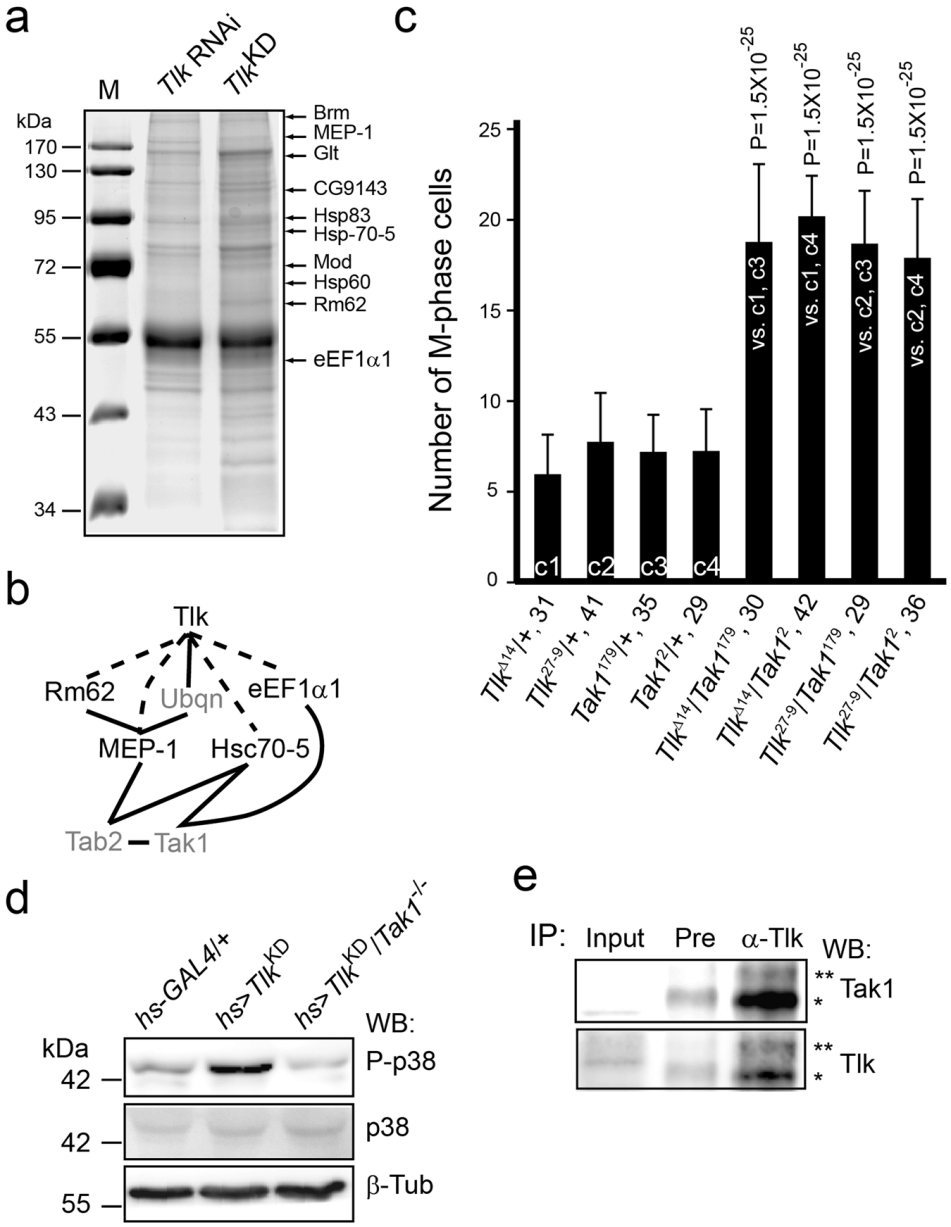
Tlk works with Tak1 to increase P-p38 level. (**a**) Protein extracts were prepared from the cold-shocked *hs* > *Tlk* RNAi (*Tlk* RNAi; as a wild type control) and *hs* > *Tlk*^KD^ (*Tlk*^KD^) embryos as that described in Fig. 4. Proteins were immunoprecipitated by anti-Tlk antibody, separated in a 10% SDS polyacrylamide gel and visualized by coomassie blue staining. Proteins in the gels were identified by mass spectrometry. The proteins marked on the right are examples of those that are uniquely detected in *hs* > *Tlk*^KD^ embryos. (**b**) A protein-protein interaction map exhibits that Tlk might work with Tak1. eEF1*α*1, Hsc70-5, MEP-1 and Rm62, identified from the co-IP experiment (dotted lines, Supplementary Table S2) and genetically confirmed their interactions with *Tlk* by the second screening experiments. Solid lines and proteins labeled in gray represent the protein-protein interactions found in BioGRID. (**c**) Increased number of M phase cell in eye disc transheterozygous for *Tlk* and *Tak*1 mutants. The experiment was performed as that in Fig. 3. Abbreviated genotypes of the eye discs are shown, followed by the number of eye disc **(**biological replicate) measured (mean ± s.d.) (see Immunostaining in Methods). Controls and tests are marked in the bar graph as described in Fig. 3. The statistical analysis using one way ANOVA test is described in Fig. 1a. The P value is shown above each test bar. (**d**) P-p38 level is reduced in embryos lacking *Tak1* activity. Embryos were collected from females of *hs*-*GAL4* or *Tak1*^2^; *hs*-*GAL4* crossed with males of *w*^1118^ (*hs*-*GAL4*/+), *UAS*-*Tlk*^KD^ (hs > *Tlk*^KD^) or *Tak1*^179^; *UAS*-*Tlk*^KD^ (hs > *Tlk*^KD^/*Tak1*^−/−^) for detection of P-p38. Quantity of β-Tub serves as a loading control. (**e**) Tak1 associates with Tlk. The co-IPed protein complex, crosslinked by BMH, was separated in a SDS agarose/polyacrylamide gel for detection of both Tak1 and Tlk by western blotting. The PVDF membrane was used to detect Tak1 first and then stripped for detection of Tlk. Two and half percent of crosslinked embryonic extract used for the co-IP serves as “Input” control. The protein complexes containing both Tak1 and Tlk are indicated by * and **.

Since the p38 activity is increased by environmental insults^8^, it is reasonable to search for proteins that associate with Tlk^KD^ when under stress. Proteins were extracted from *hs* > *Tlk* RNAi and *hs* > *Tlk*^KD^ embryos cold shocked at 12 °C for 25 min and co-immunoprecipitated (co-IP) by an anti-Tlk antibody. The co-IPed proteins were identified by mass spectrometry (Fig. 5a). The co-IP experiment was repeated four times. The criteria for candidate selection was that proteins were uniquely associated with Tlk^KD^ and were repeatedly identified for at least three times. A total of 71 candidates were obtained (Supplementary Table S2). None of them was a protein kinase or phosphatase. These proteins were then utilised to search for their interaction with kinases ASK1, MKK3/6, MLKs and TAK1 that are known to phosphorylate p38^28^. From the database search, followed by the second screening experiment mentioned above, six proteins likely linking Tlk to Tak1were found. These proteins were eEF1*α*1, Hsc70-5, MEP-1, Rm62, Tab2 and Ubqn (Fig. 5b and Supplementary Fig. S5).

We performed three sets of experiment to verify the relationship between Tlk and Tak1. The dosage dependent genetic interaction experiment was first carried out to show whether *Tlk* genetically interacts with *Tak1* to prolong the G2 phase. The results showed that the number of M phase cell in the 4–6 rows of neuronal clusters in eye disc transheterozygous for *Tlk* and *Tak1* mutants ranged from 18.0 ± 3.2 to 20.0 ± 2.2, whereas those in eye disc heterozygous for either *Tlk* or *Tak1* mutant ranged from 6.1 ± 2.1 to 7.8 ± 2.6 (Fig. 5c). The difference was significant, indicating that the genetic interaction between *Tlk* and *Tak1* was required for the G2 delay. The western blotting was then used to show whether this genetic interaction increases P-p38 level. Embryos from *Tak1*^2^; *hs*-*GAL4* females crossed with *Tak1*^179^; *UAS*-*Tlk*^KD^ males (*hs* > *Tlk*^KD^/*Tak1*^−/−^) were cold shocked. The results showed a lower P-p38 level in embryos lacking *Tak1* activity than that in *hs* > *Tlk*^KD^ embryos (Fig. 5d), indicating that *Tak1* activity was required for elevating P-p38 level. Finally, immunoprecipitation with anti-Tlk antibody was used. The results showed that Tak1 and Tlk were present in the same cross-linked protein complex (Fig. 5e). In conclusion, these results supported that the inactive Tlk associates with Tak1 to elevate P-p38 level when under stress.

### *Hsc70-5* and *eEF1α1* function together with *Tlk* and *Tak1* to delay the G2 phase when under stress

**Figure 6:**
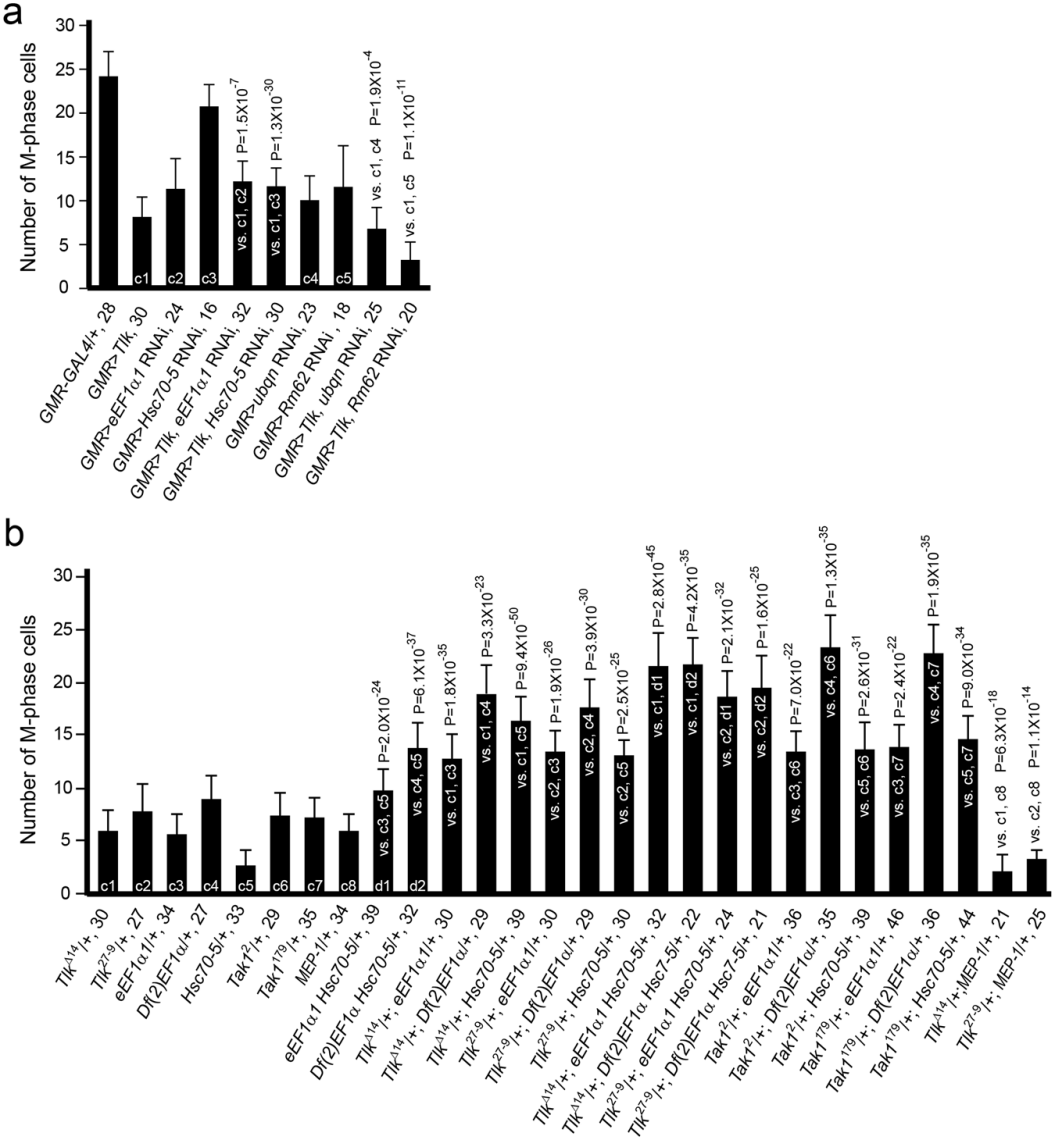
*Tlk* cooperates with *eEF1α1*, *Hsc70-5* and *Tak1* to increase the *p38* activity, leading to the G2 delay. (**a**) The G2 delay induced by *Tlk* overexpression was suppressed by knock-down of either *eEF1α1* or *Hsc70-5* activity. The number of M phase cell in the 4–6 rows of neuronal clusters was obtained as that described in Fig. 3. Females of *GMR* > *Tlk*^T2^ were crossed with males carrying a *UAS*-RNAi transgene that knocks down *eEF1α1*, *Hsc70-5*, *Rm62*, *ubqn* or *MEP-1* gene activity. Abbreviated genotypes of the eye discs are shown, followed by the number of eye disc **(**biological replicate) measured (mean ± s.d.) (see Immunostaining in Methods). Controls and tests are marked in the bar graph as described in Fig. 3. The controls are eye disc with either *Tlk* overexpression or RNAi knockdown whereas the tests are eye disc with both *Tlk* overexpression and RNAi knockdown. The statistical analysis using one way ANOVA test is described in Fig. 1a. The P value is shown above each test bar. Although *GMR* > *Tlk*^T2^; *MEP-1* RNAi/+ larvae were not sufficient for statistical analysis (data not shown), the results of the genetic interaction studies showed an enhancement of the G2 delay as shown in panel b. (**b**) *eEF1α1*, *Hsc70-5*, *Tak1 and Tlk* function together to increase p38 activity, as shown by the increased number of M phase cell in eye discs transheterozygous for two or three gene mutants among them. Abbreviated genotypes of the eye discs are shown, followed by the number of eye disc **(**biological replicate) measured (mean ± s.d.) (see Immunostaining in Methods). Controls and tests are marked in the bar graph as described in Fig. 3. The numbers of M phase cell in eye discs with genotypes of *eEF1α1 Hsc70-5*/+ and *Df*(*2*)*EF1α1 Hsc70-5*/+ are used as controls (d1 and d2) as well as tests (“vs. c3, c5” and “vs. c4, c5”). The statistical analysis using one way ANOVA test is described in Fig. 1a. The P value is shown above each test bar.

The epistatic experiment was performed to clarify the relationship of *eEF1α1*, *hsc70-5*, *MEP-1*, *Rm62*, and *ubqn* with *Tlk* (Fig. 5b). The results showed that knockdown of *eEF1α1* or *Hsc70-5* activity suppressed the G2 delay, whereas knockdown of the other gene activities did not (Fig. 6a). Thus, both *eEF1α1* and *Hsc70-5* mutants were assessed along with *Tlk* and *Tak1* mutants in the dosage dependent genetic interaction experiment. In the 4–6 rows of neuronal clusters, the numbers of M phase cell in eye disc transheterozygous for at least two mutants (from 9.8 ± 2.0 to 23.4 ± 3.1) were significantly greater than those in eye disc heterozygous for only one mutant (from 2.8 ± 1.5 to 9.1 ± 2.3) (Fig. 6b). In conclusion, *Tlk*, *eEF1α1*, *Hsc70-5* and *Tak1* function together, likely in a protein complex, to elevate *p38* activity, which in turn prolongs the G2 phase and allows cells to complete damage repairs in eye disc under stress.

## Discussion

**Figure 7:**
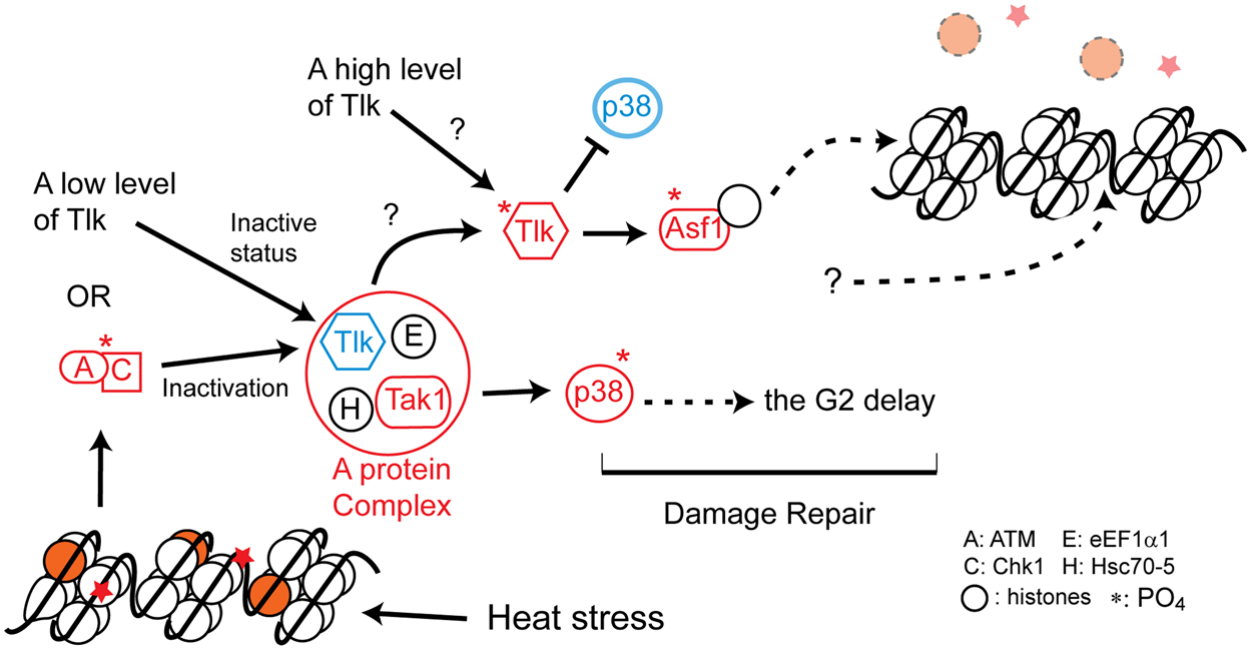
*Tlk* differentially modulates the *p38* activity when under stress. A model is proposed to explain the role of Tlk in the G2/M transition. Tlk remains in an inactive status when its level is low. The inactive Tlk associates with eEF1*α*1 (E), Hsc70-5 (H) and Tak1 to increase the *p38* activity that causes the G2 delay. Alternatively, a stress induces DNA and/or histone damage, represented by red stars and orange circles, respectively, that triggers dATM (A)/Dchk1 (C) activity to inactivate Tlk kinase activity. When under stress and/or at a high Tlk level, kinase activity of Tlk can be activated by unknown mechanisms, indicated by question marks (?). The activated Tlk then decreases the *p38* activity and increases *asf1* activity to promote the G2 recovery. Thus, Tlk acts as a guardian of genome integrity. Asterisks indicate the phosphate group. All active and inactive proteins are marked in red and blue, respectively.

The goal of this study is to clarify the role of Tlk in the G2/M transition. We showed widened distribution of CycA, indicating that *Tlk* overexpression prolongs the G2 phase. The results from the genetic interaction studies indicated that *Tlk* increases the *p38* activity, independent of Tlk kinase activity. The inactive Tlk associates with eEF1*α*1, Hsc70-5 and Tak1 in a protein complex to elevate p38 activity when under stress. Interestingly, our results also showed that a higher level of wild type Tlk reduces P-p38 level, consistent with that *Tlk* functions in the G2 recovery^3–5^. These results demonstrated that Tlk plays a dual function to prolong the G2 phase and to promote the G2 recovery via modulation of P-p38 levels (Fig. 7). This outcome supports the hypothesis that human TLKs act as a guardian of genome integrity^29^.

When under cold shock, the level of Tlk was similar in embryo from all four genotypes (12 °C in Supplementary Fig. S3c), consistent with no expression from *hsp* genes^25^ since the *hsp70* promoter is in *hs*-*GAL4* and *UAS*-*Tlk* transgenes. However, the level of P-p38 was significantly increased in *hs* > *Tlk*^KD^ embryo whereas it was significantly decreased in *hs* > *Tlk*^T2^ and *hs* > *Tlk*^#0^ embryo (Fig. 4). Our explanation lies in transcriptional leakage from the *hsp70* promoter at 25 °C, resulting in the higher level of Tlk in *hs* > *Tlk* embryos when compared to control embryo (25 °C in Supplementary Fig. S3c). The exceeding wild type Tlk or Tlk^KD^ is thus expressed from the *UAS*-*Tlk* transgenes. When embryo encounters cold shock, the stress likely triggers the formation of Tlk^KD^ associated protein complex (Fig. 7) that increases P-p38 level significantly. On the other hand, the same stress likely causes mild Tlk phosphorylation in *hs* > *Tlk*^T2^ and *hs* > *Tlk*^#0^ embryos, followed by an autophosphorylation^30^ due to over-threshold level of Tlk. Thus, Tlk is activated to dephosphorylate P-p38. In control embryo *hs*-*GAL4*+, endogenous Tlk may not reach the threshold level for autophosphorylation, thus Tlk remains at a slightly phosphorylated form which has no effect on the level of P-p38. The residual P-p38 is likely from other stress response kinases. In conclusion, Tlk plays a dual function in modulating p38 activity dependent on its level when under stress.

The dual function of wild type Tlk plus the time course of gene expression are used to illustrate the Tlk role in the G2/M transition in eye discs when under stress. Based on the time course, *Tlk* expression initiates at the middle of MF, therefore, Tlk protein level should be low in this region. It is possible that more inactive form of Tlk exists at the 1–4 rows of neuronal clusters. The inactive Tlk associates with Tak1 to increase p38 activity that prolongs the G2 phase. Tlk protein is slowly and gradually accumulated to a higher level. As described above, the Tlk kinase activity is increased by autophosphorylation when the Tlk level is over the threshold at the 6 row of neuronal clusters. The activated Tlk executes two different functions after it is released from the protein complex. The first is to dephosphorylate P-p38 that reduces p38 activity at the 6 row of neuronal clusters and after. The second is to activate Asf1 that promotes the G2 recovery^3–5^ (Fig. 7). A potential pitfall regarding the Tlk activation is that stress is required according to the very low P-p38 level in *hs* > *Tlk*^#0^ embryo without cold shock (compared 25 °C with 12 °C in Fig. 4). Mechanic stress can be used to solve this pitfall. Mechanic stress in follicle cells is generated when the shape of an egg chamber changes from sphere to ellipsoid at stages 8 and after^31^. The stress not only increases Tlk level, also induces translocation of Tlk from nucleus to cytoplasm (Supplementary Fig. S7). A similar scenario is observed in eye disc. The apical region of a row of epithelial cells inside MF is constricted, which resembles the narrow apical region of follicle cells that lack *Tlk* activity^32^. This supports that the overexpressed Tlk is inactive in the 1–4 rows of neuronal clusters. By the 5 row of neuronal clusters, the cell shape changes back to columnar^33^. Based on the effect of mechanic stress on Tlk (Supplementary Fig. S7) and the requirement of *Tlk* activity to maintain the columnar shape of follicle cells^32^, mechanic stress likely activates Tlk activity. The autophosphorylation further increases the Tlk activity when its protein level is high. The activated Tlk decreases the p38 activity and increases Asf1 activity, leading to most cells completing their cell division in the 7–10 rows of neuronal clusters^21^.

The prolonged G2 phase is important for maintaining genome stability when under stress. As described above, heat shock was applied to prolong the G2 phase based on the fact that heat shock is able to induce double stranded breaks on chromosome DNA^24^. There might be an alternative mechanism to inactivate Tlk temporally when DNA damage occurs. It is well known that kinase activities of ATM and CHK1 are activated in responding to DNA damage. Then, the activated ATM and CHK1 transiently inactivate TLK1 and TLK2^16^. Therefore, the inactivated Tlks work with Tak1 to increase p38 activity and to prolong the G2 phase transiently in order to provide sufficient time to complete damage repair (Fig. 7). This is similar to the finding that p38 is activated by TAK1 associated with the ATM/NEMO/RIP1 complex when human cell encounters genotoxic stress^34^. More importantly, the activated p38 phosphorylates p53 protein that maintains genome integrity^35^.

It is not a unique finding that the inactive Tlk acts as a coactivator. Tlk has been found to serve as a coactivator of Aur-B in either *C*. *elegans* or *T*. *brucei*, independent of its kinase activity^13,36^. Moreover, alternative functions have been reported for several other kinases as well, such as RAF, ULK3, KSR1 and EGFR^37–40^. For EGFR, activated receptor turns on the RAS/RAF/ERK cascade, whereas inactive receptor involves in the initiation of autophagy through its association with a protein complex containing LAPTM4 and SEC5^39^.

The p38 activity increased by the inactive Tlk/Tak1 complex is able to ensure survival of fly under stress. This differs from the severe rough-eye phenotype caused by *Tak1* overexpression alone, in which JNK activity is activated and induces apoptosis^41^. Although *Tlk* overexpression induces cell death, the regulatory pathway is not through apoptosis^42^. Thus, the difference may result from alternative functions of Tak1 when it works with different partners. In human cell, TAK1 is a component in the TGF-β pathway. The TGF-β receptor is activated once it is bound by TGF-β and recruits TAK1 via TRAF6 and MAD7 to activate TAK1, leading to activation of JNK^43^. On the other hand, when cell is stimulated by cytokine IL-1, TAK1 associates with TRAF6 and TAB2/3 to activate IκB kinase that facilitates the inflammatory response^44^. These indicate that Tak1 functions in immunity and protection, consistent with the roles of p38^10,11,45^. In summary, the inactivated Tlk/Tak1 complex is able to increase survivability through activation of p38 when fly confronts to various stresses.

By co-IP and mass spectrometry, seven heat shock proteins (Hsps) were found (Supplementary Table S2). Six of them were ruled out by the results from the second screening experiment, except Hsc70-5. Hsp60, Hsc70-3 and Hsc70-4 were excluded even though their interactions with Tak1 are found in BioGRID. The simplest interpretation is their subcellular localization based on the fact that protein must exist in the nucleus to regulate the *Cdk1* activity^46^. Besides both Hsp60 and Hsc70-5 are dominantly localized in mitochondria, Hsp60 is detected in the cytoplasm^47^, whereas Hsc70-5 co-localizes with p53 in the nucleus^48^, indicating that Hsc70-5 is a nuclear protein. However, the nuclear localization cannot be applied to the exclusion of Hsc70-3 and Hsc70-4 because these three proteins are detected in the nucleus in addition to their chaperone functions to restore the structure and function of denatured proteins in cytoplasm^49^. Alternatively, Hsc70-5 has a non-canonical function, but others have not. Wu and colleagues demonstrate that HSC70-5 inhibits the Raf/MEK/ERK activity and that other members cannot effectively replace HSC70-5 for the inhibition^50^. In addition, ATM is translocated from nucleus to cytoplasm in responding to genotoxic stress in human cell^51^. As shown in Supplementary Fig. S7, Tlk is detected in cytoplasm when under stress. Therefore, it is likely that nuclear dATM is transferred to cytoplasm and works with Dchk1 to inactivate the cytoplasmic Tlk. Hsc70-5 not only associates with the inactivated Tlk for maintaining the inactive status of Tlk, also recruits eEF1*α*1 to reinforce the formation of the Tlk/Tak1 protein complex that increases the P-p38 level. P-p38 enters nucleus to inhibit Cdk1 activity^52^, leading to the G2 delay. These also explain why Hsc70-5 works with inactive Tlk, but not other members. This hypothesis and the molecular mechanism for activating Tlk activity need further investigation.

## Methods

### Fly stocks and genetics

Three different *UAS*-*Tlk* transgenic lines, T2, #0 and KD (kinase dead), and *GMR*-*GAL4* are described in Supplementary Table S1. Two lines, #0 and KD, were generously provided by Dr. Karch^18^. A line carrying both *GMR*-*GAL4*^53^ and *UAS*-*Tlk*^T2^ was generated, *GMR* > *Tlk*, in which *Tlk* overexpression is indirectly driven by Glass activator (Supplementary Table S1). Fly lines *cdc2*^E1-23^, *cycB*^2^, *polo*^1^, *UAS-stg*, *Tak1*^2^, *Tak1*^179^, *Df* (*2R*) *ED2247* (a chromosome aberration deletes 37 genes, including *eEF1α1*) were obtained from Bloomington Stock Center. Fly lines *cycA*^3^^54^, *stg*^1^^55^, *p38a*^1^, *p38a*^13^^10,11^, *UAS-p38b*^DN^ ^56^ and *Tlk*^Δ14^ ^18^ were generously provided by Drs. Vincent, Posakony, Cagan, Han, Matsumoto and Karch. A *MEP*-*1* mutant line, *y*^1^ *w*^67c23^*; P{w*^*+mC*^ = *GSV6}GS12243*/*TM3*, *Sb*^1^ *Ser*^1^, was obtained from the Kyoto Stock Center. *Tlk*^27–9^ ^21^, together with *Tlk*^Δ14^, was used to perform the dosage-dependent genetic interactions with the mutants described above. To knock down gene activities of *eEF*1*α*1, *Hsc70-5*, *Rm62* and *MEP-1*, the *UAS*-RNAi lines, *P*{*TRiP*. *HMS00917*}*attP2*, 8542R-1, 10279R-1 and *P*{*TRiP*. *HMS00540*}*attP2*, were obtained from NIG-FLY and Bloomington Stock Center. The RNAi line to knock down *ubqn* activity was provided by Dr. T. Chien (Vienna Drosophila RNAi Center #47447).

Alleles of *eEF1α1* and *Hsc70-5*, *PBac*{*SAstopDsRed*}*LL06026* and *PBac*{*SAstopDsRed*}*LL00908* abbreviated as *eEF1α1*^LL06026^ and *Hsc70-5*^LL00908^, were obtained from DGRC, Kyoto Stock Center. Both lines are homozygous lethal. Using meiotic recombination to remove the P{FRT} transgenes, some progenies were homozygous viable. *eEF1α1*^LL06026^ failed to complement the *Df* (2*R*) *ED2247* deletion mutant, indicating that it is a *eEF1α1* hypomorph (definition shown at the end of Supplementary Table S1). To *Hsc70-5*^LL00908^, two genetic results support that *Hsc70-5*^LL00908^ is a hypomorph: there were no *eEF1α1*^06026^ *Hsc70-5*^LL00908^/*eEF1α1*^06026^ and a few *eEF1α1*^06026^ *Hsc70-5*^LL00908^/*Hsc70-5*^LL00908^ progenies; and there were 44.4% viable progenies from *Hsc70-5*^LL00908^ females crossed with *Hsc70-5*^k04907^/*CyO* males (Bloomington Stock Center).

All fly crosses and genotypic information are listed in Supplementary Table S1. A conceptual illustration of the dosage dependent genetic interaction experiment is also shown at the end of this table.

My laboratory was approved by Bureau of Animal and Plant Health Inspection and Quarantine, Council of Agriculture, Executive Yuan, Taiwan, to perform fly experiments. The experiments were informed to the Institutional Animal Care and Use Committee in National Yang-Ming University.

### Immunostaining

Cell division in the SMW is synchronized. Therefore, it is a nice model system to study function of genes in mitosis. During morphogenesis of *Drosophila* adult eye, the apical region of a row of epithelial cells synchronously constricts to form MF. The MF sweeps from the posterior to the anterior of the eye disc. In the middle of MF, an array of the R8 photoreceptors is first specified and serves as nuclear centers to instruct neighboring cells to become other photoreceptors that form neuronal clusters. By the 5 row of neuronal clusters, the shape of epithelial cells resumes to columnar^33^. Non-photoreceptor cells in SMW synchronously divide once. Most cells complete their divisions at the 4–6 rows of neuronal clusters, where we counted the number of M phase cell. A few cells finish their division at the 7–10 rows^22^.

The late third instar larvae with or without heat shock at 37 °C for 1 hour were harvested for dissecting eye-antennal discs. The immunostaining of eye disc was performed as described by Li *et al*.^21^. To reduce stress coming from food competition among larvae, the number of eggs laid on fly food was limited to <100 eggs. Eye discs were dissected from late 3^rd^ instar larvae and put into paraformaldehyde fixative within 10 min. The primary antibodies were anti-phosphohistone H3 (1:200; Upstate Biotechnology #H0412), anti-CycA (1:200; DSHB #A12) or anti-CycB (1:200; DSHB #F2F4). Approximately 25 eye discs were mounted in a mounting medium (20 mM Tris-HCl pH 8.8, 50% glycerol and 2% n-propyl gallate) and viewed under a confocal microscope (Leica Model TCS-SP2 or Olympus Model FV-10)^57^. To unmask the epitope recognized by the anti-CycA antibody, the procedure of antigen retrieval was performed as described by Ino^58^. In brief, eye discs fixed by the paraformaldehyde fixative were incubated in 10 mM sodium citrate and 1 mM EDTA, pH 6.0 overnight prior to heating at 90 °C for 15 minutes for retrieving the epitope.

Collection of data for statistical analysis is described as followings. The pair of eye disc in one larva is considered as two independent tissues. Late 3^rd^ instar larvae from each fly cross (see parents in Supplementary Table S1) were used to perform the immunostaining of eye disc twice approximately 4 days apart with larvae from different vials. If data were insufficient for statistical analysis, the cross and immunostaining was repeated once. Stained eye discs without distortion or disruption on mounting slides were used for counting and measurement of M phase cell and width of protein distribution, respectively. Individual eye disc was counted or measured only once, thus no technical replicate. For example, “*p38a*^1^/+, 41” in Fig. 3b means that 41 eye discs accumulated from 3 batches of *p38a*^1^/+ larvae were used to count M-phase cell one by one. Each eye disc is one biological replicate, therefore, the biological replicate n is 41 in this case. For statistical analysis, one way ANOVA test was used among 3 different genotypes, whereas two tailed analysis of Student’s *t* test was used between 2 genotypes.

### Western blotting

To determine the level of Tlk and phospho-p38 proteins (P-p38) and p38, approximately 1000 virgin females of w*, *UAS*-*Tlk*^T2^, *UAS*-*Tlk*^#0^ and *UAS*-*Tlk*^KD^ were crossed with *hs*-*GAL4* males for collecting 0–6 hour old embryos. The collected embryos were aged for 12 hours and then incubated at 12 °C for 25 minutes and lysed in RIPA buffer (20 mM Tris-HCl pH 7.5, 150 mM NaCl, 1% Triton X-100, 0.1% SDS, 1 mM EDTA, 20 mM NaF, 50 mM β-gycerophosphate, 5 mM Na_3_VO_4_ and Roche protease inhibitor cocktail). Proteins were separated in 10% SDS polyacrylamide gels and then transferred to PVDF membrane for detecting proteins using anti-p38 (1:1000, Millipore clone 2F11 MABS1754), anti-P-p38 (1:1500, Cell Signaling Technology #9211), anti-Tak1 (1:2000, Novus Biologicals #NBP2-20557), anti-Tlk (1:20,000 produced by my lab or 1:2000 provided by Dr. Karch) or anti-β-Tub (1:5000; DSHB #E7) antibodies. To detect P-p38, embryonic extracts were immediately loaded onto SDS polyacrylamide gels once they are ready and then transferred onto PVDF membranes after electrophoresis. The specificity of anti-Tak1 antibody was enhanced by preabsorption with PVDF membrane that contained 3-4 lanes of proteins extracted from embryos homozygous for *Tak1*^2^ and then PVDF membranes without proteins around 70 kDa. After incubation with proper secondary antibody, the Chemiluminescent Assay kit (ECL^TM^ Primer Western Detection Reagent, Western Lighting^TM^, Blossom Biotechnologies, Inc, Taiwan) was used to detect the proteins using a Fujifilm LAS4000 Luminescent Imaging System^21^. Intensity of the bands was measured using Image-J.

### Co-immunoprecipitation

Plasmid for expressing GST-Tlk fusion protein was generously provided by Dr. Karch^18^. Both the production and affinity purification of anti-Tlk antibody were carried out by GeneTex Inc. Its specificity was determined by western blotting (Supplementary Fig. S3a) and immunohistochemistry as described in Yeh *et al*.^32^.

The collection and treatment of *Drosophila* embryos was as described in the western blotting section. Procedure of co-IP described in Thermo Scientific Co was used. The co-IPed proteins were separated in 10% SDS polyacrylamide gel and stained by coomassie blue. To each lane, twelve gel blocks were consecutively taken according to the banding pattern. Proteins in each gel block were identified using mass spectrometry (Proteomics Research Center, National Yang-Ming University).

To determine whether Tlk and Tak1 exist in the same protein complex, proteins in the embryonic extract were cross-linked by 35 μg/ml of bismaleimidohexane (BMH, Thermo Scientific Co.#22330) using the procedure described by Liu *et al*.^59^. To reduce protein non-specifically bound by antibody, both pre-immune and anti-Tlk antibody were pre-absorbed by 12–18 hours of wild-type embryos. Protein complex was co-IPed using the pre-absorbed antibodies. The co-IPed protein complex was separated in 0.7% agarose/3.4% SDS polyacrylamide gel with 8% SDS polyacrylamide gel as a cushion^60^ and detected by western blotting with anti-Tlk and anti-Tak1 antibodies.

## Electronic supplementary material

Supplementary file
