# Supplementary material for: Inactive Tlk associating with Tak1 increases p38 MAPK activity to prolong the G2 phase
Source: Sci Rep. 2019 Feb 13;9:1885. doi: 10.1038/s41598-018-36137-1 (PMC6374402; doi:10.1038/s41598-018-36137-1)
Supplement: Supplementary file 1 — Supplementary file [file 41598_2018_36137_MOESM1_ESM.pdf]

Inactive Tlk associating with Tak1 increases p38 MAPK activity to prolong the G2 phase

Gwo-Jen Liaw and Chuen-Sheue Chiang

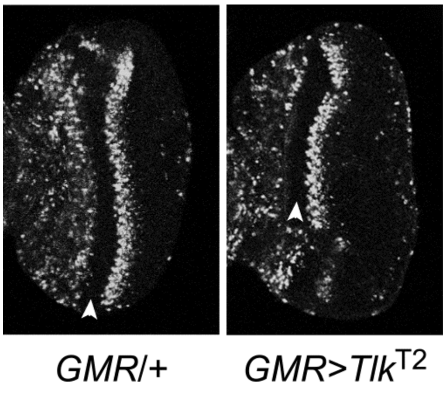


Figure S1. *Tlk* overexpression does not affect progression of S phase.
To reveal whether *Tlk* overexpression affects progression of the S phase at the right side of morphogenetic furrow (MF), indicated by arrow heads, the bromodeoxyuridine (BrdU) labeling of eye discs was performed as described by Wolf[1]. Eye-antennal discs with *GMR-GAL4*/+ (*GMR*/+) or *GMR*>*Tlk*$^{T2}$ were dissected from late 3$^{rd}$ instar larvae and incubated in 75 μg/ml BrdU dissolved in 1X PBS. The incorporated BrdU was detected by anti-BrdU antibody and visualized by confocal microscope. Results indicated that *Tlk* overexpression does not affect the S phase progression.

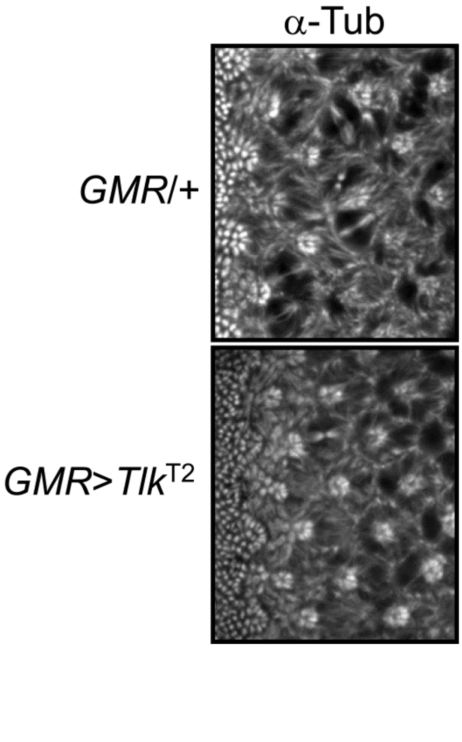

Fig. S2. *Tlk* overexpression causes a subtle defect on microtubule morphology.

To reveal whether *Tlk* overexpression, *GMR*>*Tlk*$^{T2}$, affects microtubule, eye discs were incubated with anti-α-Tubulin (α-Tub) antibody, and observed under confocal microscope. MF is at the left. Morphology of microtubule was slightly affected by *Tlk* overexpression, consistent with the results shown by Yeh at al.[2].

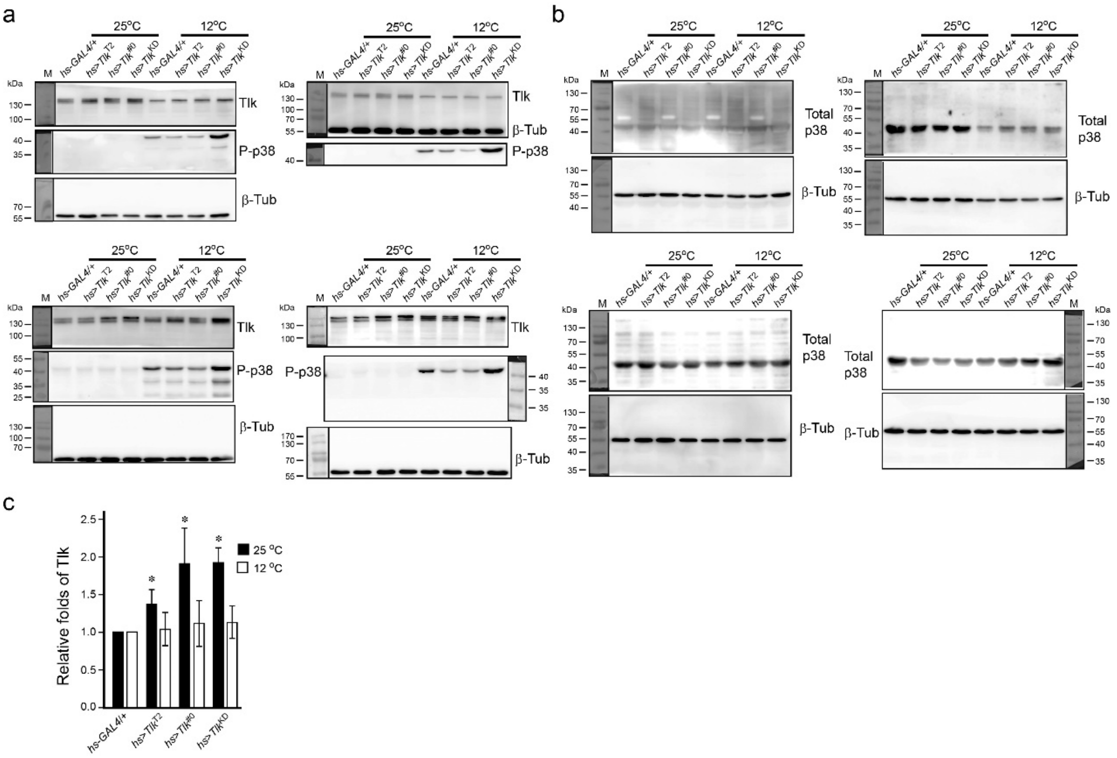


Figure S3. Images of western blotting were used to determine levels of phosphorylated p38 MAPK (P-p38) and total p38.

(**a**) High specificity of the anti-Tlk antibody prepared in our laboratory. Production of GST-Tlk fusion protein and anti-Tlk antibody is described in Methods. Western blotting was used to determine specificity of the newly produced anti-Tlk antibody (see Tlk panel, 25°C, without cold shock). According to the annotated gene products, sizes of the six putative Tlk proteins are 111, 136, 138, 139, 161 and 164 kDa. This antibody recognized Tlks with sizes of 136, 138 139 kDa, which is fewer than those recognized by antibody provided by Dr. Karch[3] (Supplementary Fig. S4a,b). The results showed that specificity of the newly produced anti-Tlk antibody is high. The specificity of this anti-Tlk antibody was also tested using immunohistochemistry[2]. In addition, protein extracts were made and loaded onto four 10% SDS gels immediately. There were 4 batches of embryonic extracts. Each PVDF membrane was divided into 2 parts around 52 kDa for detection of Tlk, P-p38, p38 and β-Tubulin (β-Tub) by western blotting. However, using the recommended buffer from the company that

contains 1X TBS containing 0.1% Tween-20 and 5% BSA, total p38 were undetectable. Abbreviated genotypes of the embryos are on the top of each panel. (**b**) The undetectable problem was solved by changing the buffer to 1X PBS containing 0.25% Tween 20, 0.25% Triton X-100 and 1% BSA. To reduce the high background as shown in the top left panel, the membranes were briefly stripped after the first incubation with the anti-p38 antibody without the checking procedure and re-incubated with the same antibody solution again. After the detection of the total p38, the membrane was stripped for detecting β-Tub to serve as loading control. N.B.: Pyronin Y, a marker for dividing membrane, is not washed away and stays on membrane after the membrane is dried, shown as 4 visible empty bands. (**c**) The bar graph shows Tlk levels in various embryos with (open bars) and without (solid bars) cold shock. Quantity of both Tlk and β-Tub were measured using Image J. Quantity of Tlk was first normalized by quantity of β-Tub. Relative folds to the control, *hs-GAL4*/+, were separately calculated, 25°C and 12°C. In the three different *hs*>*Tlk* embryos without cold shock, their quantities are significantly higher than that in the *hs-GAL4*/+ control, ranging from 1.39 ± 0.2 to 1.91 ± 0.2. Statistical significance was determined by one way ANOVA test (*: P=0.00033). In contrast, their levels are insignificantly different in these embryos with cold shock (one way ANOVA test, P=0.72).

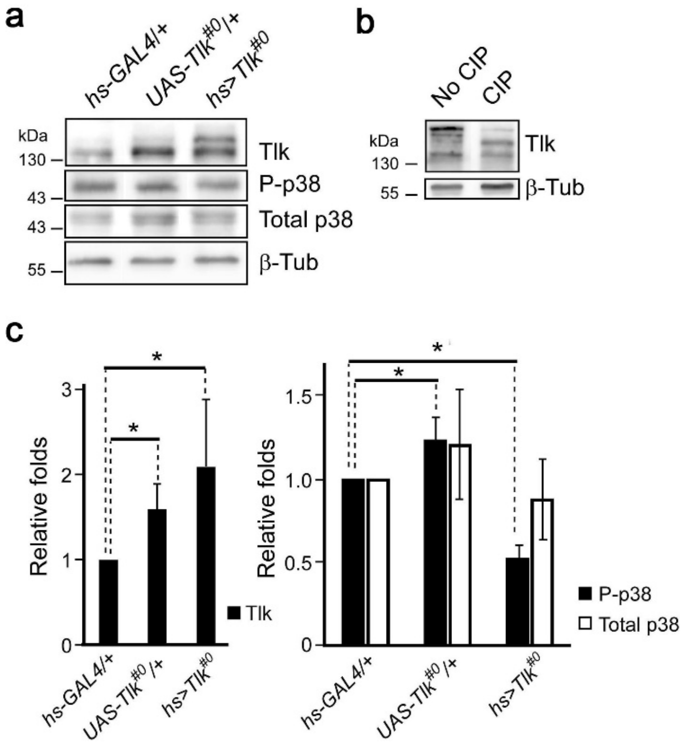

Figure S4. Wild type Tlk plays a dual function in modulating P-p38 levels, depending on Tlk levels.

The *UAS-Tlk*$^{T2}$ transgene was indirectly activated by the *GMR* enhancer to overexpress the wild type *Tlk* in the region behind morphogenetic furrow (MF), including MF. *Tlk* overexpression causes a shift from the 4-6 to the 7-10 rows of neuronal clusters where most cells complete their cell division[4]. According to the time course of gene expression, the Tlk level is expected to be low in MF and to be high after the 6 row of neuronal cluster[5]. The difference in the Tlk levels might attribute differently to P-p38 levels. To test this hypothesis, we needed a fly that expresses a Tlk level lower than that in *hs*>*Tlk* fly, and a proper experimental procedure including heat shock and room temperature incubation. The incubation provides time for Tlk accumulation to different levels between *UAS-Tlk*$^{\#0}$/+ and *hs*>*Tlk*$^{\#0}$ flies. (**a**) *hs-GAL4*/+, *UAS-tlk*$^{\#0}$/+ and *hs>tlk*$^{\#0}$ adult females were heat shocked at 38.5°C for 15 min and incubated at room temperature for 45 min. Tlk, P-p38 and total p38 were detected and their quantities were measured by western blotting and Image J. The anti-Tlk antibody provided by Dr. Karch[3] was used. The PVDF membrane for detecting P-p38 was stripped and used for detection of β-Tub as loading control. (**b**) Protein extracts from *hs>tlk*$^{\#0}$ adult females heat shocked at 38.5°C for 75 min was treated with or without calf intestine alkaline phosphatase (CIP) to reveal phosphorylated forms of Tlk. Proteins with sizes larger than 135 kDa are sensitive to the CIP treatment, indicating that these are phosphorylated Tlks. β-Tub on the same blot was detected as loading control. (**c**) Two bar graphs show relative levels of Tlk (left panel), P-p38 (right panel, solid bars) and total p38 (right panel, open bars). Data were from five independent batches of protein extracts. All values were normalized first by quantity of β-Tub and then by quantity of Tlk, P-p38 or total p38 in *hs-GAL4*/+ fly, and showed as relative folds. The statistical significance was determined using two tailed analysis of Student's *t*-test (*: $P < 0.05$). Although the Tlk levels between *UAS-Tlk*$^{\#0}$/+ and *hs*>*Tlk*$^{\#0}$ fly are insignificantly different (left panel of c), levels of the phosphorylated Tlk are higher in *hs*>*Tlk*$^{\#0}$ fly (panels a and b). Interestingly, the P-p38 level in *UAS-Tlk*$^{\#0}$/+ fly is significantly, although marginally, higher than that in the control (right panel of c). In contrast, the P-p38 level in the *hs*>*Tlk*$^{\#0}$ fly is significantly reduced by ~50%. In summary, a low and a high level of wild type Tlk differentially modulate the P-p38 levels.

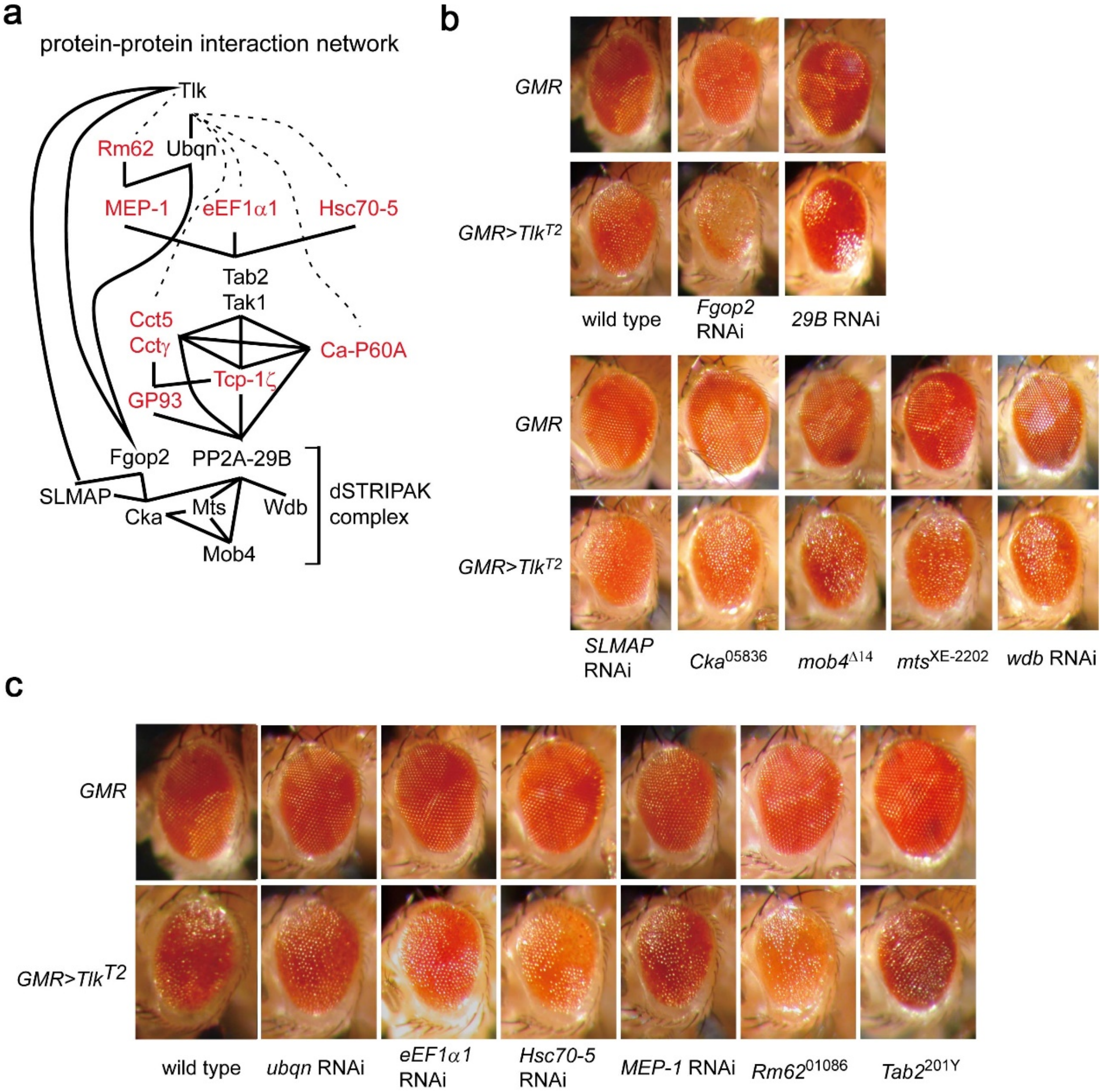


<u>Figure S5. Screening of genes that genetically interact with *Tlk*.</u>

(**a**) A mini protein-protein interaction network. To find which gene activity can modify the rough eye phenotype resulted from *Tlk* overexpression[4], *GMR*>*Tlk*$^{T2}$, a putative protein-protein interaction network was built based on information in BioGRID, indicated by black lines. This network also includes proteins marked in red, that were identified by coimmunoprecipitation and mass spectrometry (Supplementary Table S2). Dashed lines represent that the proteins indirectly interact with Tlk. (**b**) *Tlk* unlikely interacts with the *dSTRIPAK* complex. Ribeiro and colleagues identify protein components in dSTRIPAK[6], as shown at the bottom of panel a. Two components, Fgop2 (CG10158) and SLMAP (CG17494), possibly interact with Tlk (BioBRID). This suggests that Tlk regulates the phosphatase activity of dSTRIPAK. To test whether the genes, encoding the components, interact with *Tlk* genetically, females of either mutant or carrying a *UAS*-RNAi transgene were crossed with either *GMR-GAL4* (*GMR*) or *GMR*>*Tlk*$^{T2}$ males. Two different *UAS*-RNAi lines were tested in the RNAi experiments. Abbreviated genotypes of the females are

shown at the bottom. Male progenies were collected to observe morphology of the compound eye and photographed under dissecting microscope (Leica Model MZFLIII). The results showed that knockdown of either *Pp2A-29B* (*29B* RNAi) or *Fgop2* activity enhanced the rough eye phenotype, indicating that *Tlk* genetically interacts with *Fgop2* and *Pp2A-29B*. Inconsistently, knockdown or reduced activity of the remaining genes had no effect on the rough eye phenotype. To test further, adult fly transheterozygous for *Tlk* and *mob4* mutants were incubated at 37˚C. Its survivability was insignificantly different from those heterozygous for either *Tlk* or *mob4* mutant (data not shown). In summary, it is unlikely that *Tlk* regulates the *dSTRIPAK* activity. (**c**) *Tlk* genetically interacts with *eEF1αl*, *Hsc70-5*, *MEP-1*, *Rm62* and *ubqn*. As shown in panel a, four proteins were identified using coimmunoprecipitation and mass spectrometry (Supplementary Table S2) and link Tlk to the Tab2/Tak1 complex. The genetic screening, as described in panel b, was used to test whether these genes interact with *Tlk*. The results showed a weak suppression, indicating that *Tlk* interacts with these genes. N.B.: The rough eye phenotype in *GMR*>*Tlk*$^{T2}$ males in this panel is stronger than that in panel b because temperature in the fly room was higher when this batch of experiments was carried out.

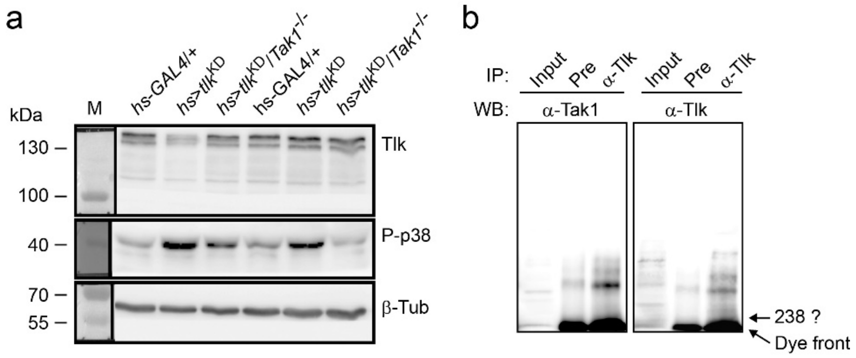


Figure S6. Inactive Tlk and Tak1 are in a protein complex that increases the P-p38 level.

(**a**) Embryos, abbreviated genotypes indicated on top, were cold shocked at 12˚C for 25 min. Proteins were extracted from the embryos, separated in a SDS gel and transferred onto PVDF membrane. The PVDF membrane was horizontally divided into 3 parts for detection of Tlk, P-p38 or β-Tubulin (β-Tub) by western blotting. The image included two batches of embryonic extracts at the left and the right, which were prepared in two consecutive days. Each batch contains *hs-GAL4*/+, *hs*>*Tlk*$^{KD}$ and *hs*>*Tlk*$^{KD}$; *Tak1*$^{-/-}$. The results indicate reduced levels of P-p38 in embryos lacking *Tak1* activity. (**b**) To determine whether Tlk and Tak1 are in a protein complex, proteins extracted from cold shocked *hs*>*Tlk*$^{KD}$ embryos and cross-linked by

bismaleimidohexane (BMH). Cross-linked protein complexes were immunoprecipitated by an anti-Tlk antibody and separated in a SDS agarose/polyacrylamide gel (see Methods). When bromophenol blue enters the cushion, 8% SDS polyacrylamide gel, the electrophoresis was stopped. Control is the preimmune serum (Pre) used to immunoprecipitate protein complex. Protein complexes were then transferred onto PVDF membrane for detection of Tak1 protein by the preabsorbed anti-Tak1 antibody (see Methods). Then, the antibodies on the membrane were stripped for detection of Tlk protein by anti-Tlk antibody, following the standard stripping protocol. IP and WB stand for immunoprecipitation and western blotting, respectively. Approximately position of an estimated 238 kDa protein marker (HiMark protein ladder, Invitrogen) is marked as 238?. Dye front indicates bottom of the gel.

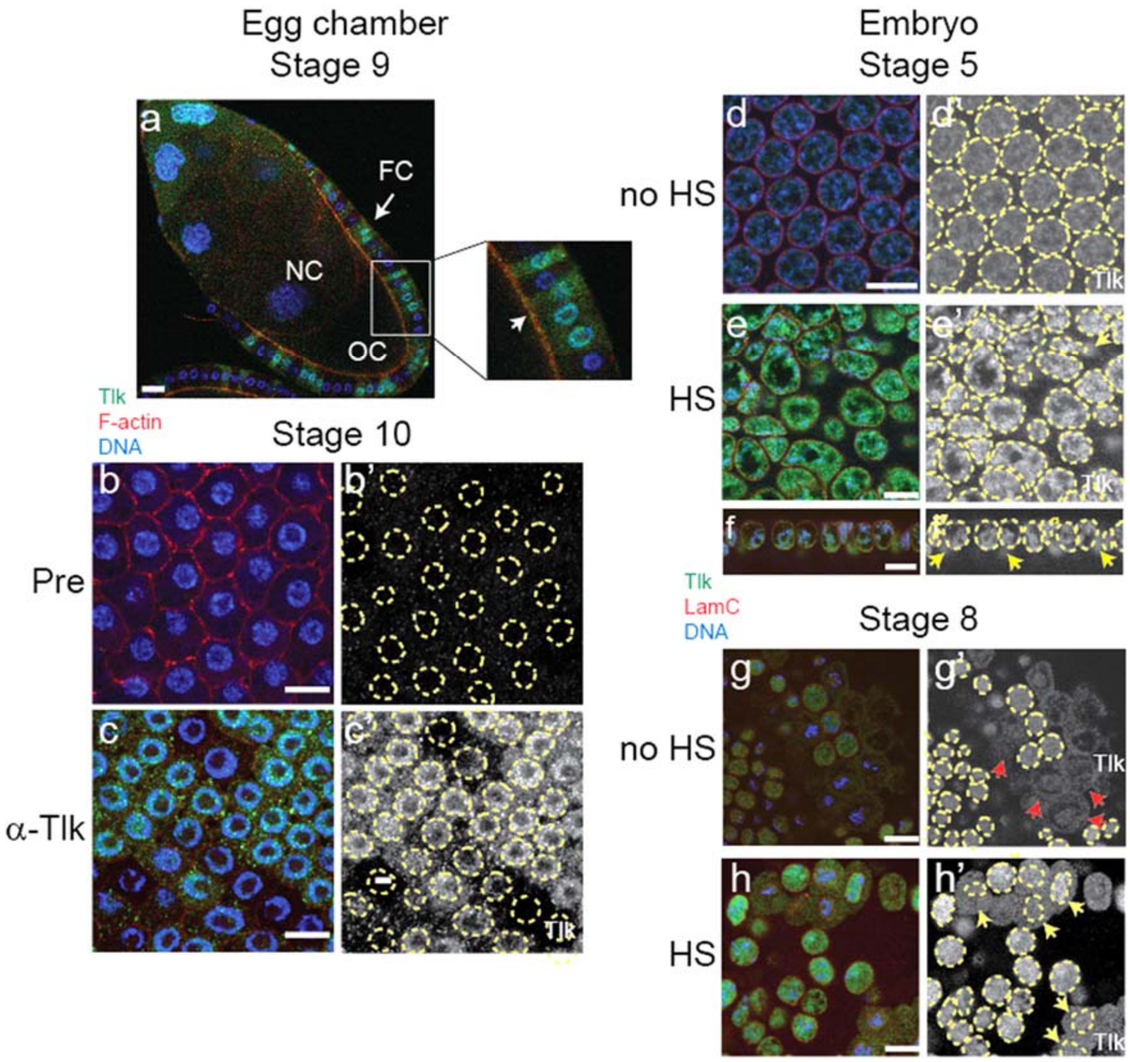


Figure S7. Stress induces translocation of Tlk from nucleus to cytoplasm.

(a-c) The affinity purified anti-Tlk antibody (Supplementary Fig. 3a) was used to

detect Tlk localization in follicle cells (FCs)[2] which surround 15 nurse cells (NC) and 1 oocyte (OC) to form an egg chamber, a unit to develop into a mature egg. (d-h) The distribution of Tlk in embryo was detected by immunohistochemistry (IHC). In stage 5 embryo, Tlk mainly localized in nuclei (d), consistent with the report by Carrera et al[3]. In stage 8 embryo, Tlk was mainly in nuclei when cells are in interphase. During M phase, Tlk was detected in entire cell, indicated by red arrows (g'). To save space, results of the embryonic IHC with the pre-immune serum (Pre) were not shown because the background was similar to that in panel b. In stage 9 and 10 egg chambers, unexpectedly, Tlk distribution in FCs differed from that in early stages of egg chamber[3] and embryo. The distribution of nuclear Tlk was not uniform in FCs along the anteroposterior axis (a). In FCs with a higher level of nuclear Tlk, a lower level of Tlk was detected in cytoplasm (a, c'). Furthermore, in a few FCs, indicated by an arrow, cytoplasmic Tlk formed a very shallow gradient from nucleus to basal region of the cells (an amplified panel in panel a). This implicated that Tlk was translocated from nucleus to cytoplasm. During oogenesis, egg chambers change their shape from a sphere (stages 6 and before) to an ellipsoid (stages 8 and after). Periodic and oscillating contractions of basal actinomyosin contribute to the morphogenetic change. Noticeably, the contraction generates mechanic stress to the FCs[7], bringing up a hypothesis that the stress induces the higher level and the cytoplasmic translocation of Tlk. To test this, Tlk in $w^{1118}$ embryos heat shocked at 37°C for 30 minutes was detected by IHC with the anti-Tlk antibody. Panel f showed that the nuclear position in stage 5 embryo was disturbed with heat shock, as different sizes of nuclei appeared on one focal plane (comparing panel e with panel d). More importantly, Tlk is detected in cytoplasm, indicated by yellow arrows (panel f'). The cytoplasmic localization of Tlk was also detected in stage 8 embryo (yellow arrows in panel h'). Furthermore, quantity of the nuclear Tlk was higher with heat shock (comparing panels e, h with d, g). These results supported that stress induces translocation of Tlk protein from nucleus to cytoplasm. Length of scale bars is 10 μm.

## References


1 Wolff, T. in *Drosophila protocols* (eds W. Sullivan, M. Ashburner, & R. S. Hawley) 201-228 (Cold Spring Harbor Laboratory Press, 2000).

2 Yeh, T. H., Huang, S. Y., Lan, W. Y., Liaw, G. J. & Yu, J. Y. Modulation of cell morphogenesis by tousled-like kinase in the Drosophila follicle cell. *Dev Dyn.* **244**, 852-865, doi:10.1002/dvdy.24292 (2015).

3 Carrera, P. *et al.* Tousled-like kinase functions with the chromatin assembly pathway regulating nuclear divisions. *Genes Dev.* **17**, 2578-2590 (2003).

4 Li, H. H., Chiang, C. S., Huang, H. Y. & Liaw, G. J. *mars* and *tousled-like kinase* act in parallel to ensure chromosome fidelity in *Drosophila*. *J Biomed Sci.* **16**, 51, doi:10.1186/1423-0127-16-51 (2009).

5 Baker, N. E. & Yu, S. Y. The EGF receptor defines domains of cell cycle progression and survival to regulate cell number in the developing *Drosophila* eye. *Cell* **104**, 699-708 (2001).

6 Ribeiro, P. S. *et al.* Combined functional genomic and proteomic approaches identify a PP2A complex as a negative regulator of Hippo signaling. *Mol Cell* **39**, 521-534, doi:10.1016/j.molcel.2010.08.002 (2010).

7 Wozniak, M. A. & Chen, C. S. Mechanotransduction in development: a growing role for contractility. *Nature reviews. Mol Cell Biol.* **10**, 34-43, doi:10.1038/nrm2592 (2009).

**Table S1.** Abbreviated genotypes of eye discs in figures 1, 2, 3, 5C and 6

| Parents (virgin females X males) | Genotypes of selected larval progenies | Abbreviated genotypes |
|---|---|---|
| **Figure 1 .** | | |
| $w^{1118}$ X *w*/*Y; *GMR-GAL4* | $w^{1118}$/*w** [a]; *GMAR-GAL4/+* | *GMR/+* |
| $w^{1118}$ X $w^{1118}$/Y; *UAS-Tlk*$^{T2}$ | *UAS-Tlk*$^{T2}$/+ | *UAS-Tlk*$^{T2}$/+ |
| $w^{1118}$; *UAS-Tlk*$^{T2}$ X *w*/*Y; *GMR-GAL4* | *UAS-Tlk*$^{T2}$/+; *GMR-GAL4/+* | *GMR>Tlk* |
| *w**; *UAS-stg* X *w*/*Y; *UAS-Tlk*$^{T2}$; *GMR-GAL4* | *UAS-Tlk*$^{T2}$/+; *GMR-GAL4/UAS-stg* | *GMR>Tlk stg* |
| *Cdk1*$^{E1\text{-}23}$/*CyO Kr>GFP* X *w*/*Y; *UAS-Tlk*$^{T2}$; *GMR-GAL4* [b] | *w*/+*; *Cdk1*$^{E1\text{-}23}$/*UAS-Tlk*$^{T2}$; *GMR-GAL4/+* | *GMR>Tlk/Cdk1*$^{E1\text{-}23}$ |
| *w**; *UAS-Cdk1* X *w*/*Y; *UAS-Tlk*$^{T2}$; *GMR-GAL4* | *UAS-Tlk*$^{T2}$/+; *GMR-GAL4/UAS-Cdk1* | *GMR>Tlk Cdk1* |
| *CycB*$^{2}$/*CyO Kr>GFP* X *w*/*Y; *UAS-Tlk*$^{T2}$; *GMR-GAL4* | *w*/+*; *CycB*$^{2}$/*UAS-Tlk*$^{T2}$; *GMR-GAL4/+* | *GMR>Tlk/CycB*$^{2}$ |
| *CycA*$^{3}$/*TM3 Sb*$^{1}$ *Kr>GFP* X *w*/*Y; *UAS-Tlk*$^{T2}$; *GMR-GAL4* | *UAS-Tlk*$^{T2}$/+; *GMR-GAL4/CycA*$^{3}$ | *GMR>Tlk/CycA*$^{3}$ |
| *stg*$^{1}$/*TM3 Sb*$^{1}$ *Kr>GFP* X *w*/*Y; *UAS-Tlk*$^{T2}$; *GMR-GAL4* | *UAS-Tlk*$^{T2}$/+; *GMR-GAL4/Stg*$^{1}$ | *GMR>Tlk/stg*$^{1}$ |

*GMR-GAL4* and *UAS-Tlk* are transgenes in transgenic flies. GMR is a multiple repeated enhancers bound by Glass transcription factor that activates *GAL4* expression in the region behind morphogenetic furrow (MF) of eye disc. A transgenic line, *UAS-Tlk*$^{T2}$, carries a *P*{$w^{+MC}$=*UAST-Tlk*} transgene containing the *Tlk* coding region in GH07910 EST clone. When *GMR-GAL4* and *UAS-Tlk* transgenes co-exist in fly genome, GAL4 activator driven by *GMR* binds to *UAS* and activates *Tlk* expression in the region behind MF. Therefore, *Tlk* is indirectly activated by *GMR*, designated as *GMR>Tlk*. T2 is an allelic name of a *UAS-Tlk* transgenic fly with a different chromosomal location from that in #0 or KD. KD represents a mutation in the ATP binding domain, resulting in that kinase activity of Tlk is not activated. GFP is a marker used to select larvae under fluorescence dissecting microscope. For example, progenies from parent, *CycB*$^{2}$/*CyO Kr>GFP* crossed with *w**; *UAS-Tlk*$^{T2}$; *GMR-GAL4*, have two different genotypes, *CycB*$^{2}$/*UAS-Tlk*$^{T2}$; *GMR-GAL4/+* (GFP$^{-}$) and *UAS-Tlk*$^{T2}$/*CyO Kr>GFP*; *GMR-GAL4/+* (GFP$^{+}$). GFP$^{-}$ larvae are picked for eye disc dissection, in which *Tlk* is overexpressed in eye disc with reduced *CycB* activity (≥50% of the wild type activity). $w^{1118}$, *w** and + represent *w* mutants with allelic names as 1118 and *, and no identified mutation on this chromosome, respectively.

[a]: For space saving in the second column, *w* alleles are omitted hereafter, such as $w^{1118}$ and/or *w**. Viability of $w^{1118}$/*w** fly is similar to that of wild type fly, Oregon R. It is often used as a wild type control. The asterisk represents that the *w* allele is uncertain because balancer lines, such as *CyO* or *TM3*, are regularly juvenilized using different *w* alleles and then re-set up the balancer lines. The *w* allele in the newly established lines remains unidentified.

[b]: Adult progenies homozygous for *w**, *UAS-Tlk*$^{T2}$ and *GMR-GAL4* is semi lethal, approximately 30% for *TM6B*/+. The homozygous males can be obtained from parents of *w** (1); *UAS-Tlk*$^{T2}$/*CyO* (2); *GMR-GAL4/TM6B Tb*$^{1}$ *Hu*$^{1}$ *e*$^{1}$ (3) for performing the crosses shown in the first column. The number in parentheses stands for the first, second and third chromosomes. Y represents the Y chromosome. Different chromosomes are separated by semicolon.

**Figure 2**

| | | |
|---|---|---|
| $w^{1118}$ X $w^*$/Y; $p38a^1$ | $p38a^1$/+ | $p38a^1$/+ |
| $polo^1$/*TM3* $Sb^1$ *Kr>GFP* X $w^*$/Y; *UAS*-$Tlk^{T2}$; *GMR-GAL4* | $w^*$/+;*UAS*-$Tlk^{T2}$/+; *GMR-GAL4*/$polo^1$ | *GMR>Tlk*/$polo^1$ |
| $w^*$; *UAS*-$polo^{T182D}$ X $w^*$/Y; *UAS*-$Tlk^{T2}$; *GMR-GAL4* | *UAS*-$Tlk^{T2}$/+; *GMR-GAL4*/*UAS*-$polo^{T182D}$ | *GMR>Tlk* $polo^{T182D}$ [c] |
| $w^*$ *UAS*-$p38b^{DN}$/*FM7 act-GFP* X $w^*$/Y; *UAS*-$Tlk^{T2}$; *GMR-GAL4* | $w^*$ *UAS*-$p38b^{DN}$/ $w^*$; *UAS*-$Tlk^{T2}$/+; *GMR-GAL4*/+ | *GMR>Tlk* $p38b^{DN}$ [c] |
| $w^*$; $p38a^1$ X $w^*$/Y; *UAS*-$Tlk^{T2}$; *GMR-GAL4* | *UAS*-$Tlk^{T2}$/+; *GMR-GAL4*/$p38a^1$ | *GMR>Tlk*/$p38a^1$ |
| $w^*$; *UAS-p38a* X $w^*$/Y; *UAS*-$Tlk^{T2}$; *GMR-GAL4* | *UAS*-$Tlk^{T2}$/+; *GMR-GAL4*/*UAS-p38a* | *GMR>Tlk p38a* |
| $w^*$; *UAS-p38a* X $w^*$/Y; *GMR-GAL4* | *GMR-GAL4*/*UAS- p38a* | *GMR>p38a* |
| [c]: T182D is a dominant active mutation of *polo* with Thr at position182 substituted by Asp. DN is a dominant negative mutation of *p38b*. | | |
| **Figure 3** | | |
| $w^{1118}$ X Oregon R/Y | $w^{1118}$/+ | $w^{1118}$/+ |
| $w^{1118}$ X $w^*$/Y; $p38a^1$ | $p38a^1$/+ | $p38a^1$/+ |
| $w^{1118}$ X $y^1$ $w^*$/Y; $p38a^{13}$ | $w^{1118}$/$y^1$ $w^*$; $p38a^{13}$/+ | $p38a^{13}$/+ |
| $w^{67c32}$ $Tlk^{\Delta 14}$/*FM7 act-GFP* X $w^{1118}$/Y | $w^{67c32}$ $Tlk^{\Delta 14}$/$w^{1118}$ | $Tlk^{\Delta 14}$/+ [d] |
| $w^{1118}$ $Tlk^{27\text{–}9}$/*FM7 act-GFP* X $w^{1118}$/Y | $w^{1118}$ $Tlk^{27\text{–}9}$/$w^{1118}$ | $Tlk^{27\text{–}9}$/+ [d] |
| $w^{1118}$ $Tlk^{27\text{–}9}$/*FM7 act-GFP* X w*/Y; $p38a^1$ | $w^{1118}$ $Tlk^{27\text{–}9}$/$w^*$; $p38a^1$/+ | $Tlk^{27\text{–}9}$/+; $p38a^1$/+ |
| $w^{67c32}$ $Tlk^{\Delta 14}$/*FM7 act-GFP* X $y^1$ w*/Y; $p38a^{13}$ | $w^{67c32}$ $Tlk^{\Delta 14}$/$y^1$ $w^*$; $p38a^{13}$/+ | $Tlk^{\Delta 14}$/+; $p38a^{13}$/+ |
| $w^{67c32}$ $Tlk^{\Delta 14}$/*FM7 act-GFP* X w*/Y; $p38a^1$ | $w^{67c32}$ $Tlk^{\Delta 14}$/$w^*$; $p38a^1$/+ | $Tlk^{\Delta 14}$/+; $p38a^1$/+ |
| $w^{1118}$ $Tlk^{27\text{–}9}$/*FM7 act-GFP* X $y^1$ w*/Y; $p38a^{13}$ | $w^{1118}$ $Tlk^{27\text{–}9}$/$y^1$ $w^*$; $p38a^{13}$/+ | $Tlk^{27\text{–}9}$/+; $p38a^{13}$/+ |
| [d]: These abbreviations are also used in Figures 5C and 6B. | | |
| **Figure 5C** | | |
| $w^*$ $Tak1^{179}$ X $w^{1118}$/Y | $w^*$ $Tak1^{179}$/$w^{1118}$ | $Tak1^{179}$/+ [d] |
| $y^1$ $w^*$ $Tak1^2$ X $w^{1118}$/Y | $y^1$ $w^*$ $Tak1^2$/$w^{1118}$ | $Tak1^2$/+ [d] |
| $w^{67c32}$ $Tlk^{\Delta 14}$/*FM7 act-GFP* X $w^*$ $Tak1^{179}$/Y | $w^{67c32}$ $Tlk^{\Delta 14}$ [e]/$w^*$ $Tak1^{179}$ | $Tlk^{\Delta 14}$/$Tak1^{179}$ |

| | | |
|---|---|---|
| *w*$^{67c32}$ *Tlk*$^{\Delta14}$/*FM7 act-GFP* X *y*$^{1}$ *w* Tak1*$^{2}$/Y | *w*$^{67c32}$ *Tlk*$^{\Delta14}$/*y*$^{1}$ *w* Tak1*$^{2}$ | *Tlk*$^{\Delta14}$ [e]/*Tak1*$^{2}$ |
| *w*$^{1118}$ *Tlk*$^{27\text{–}9}$/*FM7 act-GFP* X *w* Tak1*$^{179}$/Y | *w*$^{1118}$ *Tlk*$^{27\text{–}9}$/*w* Tak1*$^{179}$ | *Tlk*$^{27\text{–}9}$/*Tak1*$^{179}$ |
| *w*$^{1118}$ *Tlk*$^{27\text{–}9}$/*FM7 act-GFP* X *y*$^{1}$ *w* Tak1*$^{2}$/Y | *w*$^{1118}$ *Tlk*$^{27\text{–}9}$/*y*$^{1}$ *w* Tak1*$^{2}$ | *Tlk*$^{27\text{–}9}$/*Tak1*$^{2}$ |
| [e]: Male larvae hemizygous for *Tlk* null mutant die at 1$^{st}$ instar. Only female larvae heterozygous for *Tlk* and *Tak1* mutants survive to adult stage. | | |
| **Figure 6** | | |
| *y*$^{1}$ *sc* v*$^{1;}$ *P{TRiP*$^{HMS00917}$}attP2 X *w**/Y; *GMR-GAL4* | *y*$^{1}$ *sc* v*$^{1}$/*w**; *GMR-GAL4*/*UAS-eEF1a1*$^{RNAi}$ | *GMR>eEF1a1*$^{RNAi}$ |
| *w**; *UAS-Hsc70-5*$^{RNAi}$ X *w**/Y; *GMR-GAL4* | *UAS-Hsc70-5*$^{RNAi}$/+; *GMR-GAL44/+* | *GMR>Hsc70-5*$^{RNAi}$ |
| *y*$^{1}$ *sc* v*$^{1;}$ *P{TRiP*$^{HMS00917}$*}*attP2 X *w**/Y; *UAS-Tlk*$^{T2}$; *GMR-GAL4* | *y*$^{1}$ *sc* v*$^{1}$/*w**; *UAS-Tlk*$^{T2}$/+; *GMR-GAL4/UAS-eEF1a1*$^{RNAi}$ | *GMR>Tlk eEF1a1*$^{RNAi}$ |
| *w**; *UAS-Hsc70-5*$^{RNAi}$ X *w**/Y; *UAS-Tlk*$^{T2}$; *GMR-GAL4* | *UAS-Tlk*$^{T2}$/*UAS-Hsc70-5*$^{RNAi}$; *GMR-GAL4*/+ | *GMR>Tlk Hsc70-5*$^{RNAi}$ |
| *w*;UAS-ubqn*$^{RNAi}$ X *w**/Y; *GMR-GAL4* | *UAS-ubqn*$^{RNAi}$/+; *GMR-GAL4/+* | *GMR>ubqn*$^{RNAi}$ |
| *w*;UAS-Rm62*$^{RNAi}$ X *w**/Y; *GMR-GAL4* | *UAS- Rm62*$^{RNAi}$/+; *GMR-GAL4/+* | *GMR> Rm62*$^{RNAi}$ |
| *w*; UAS-ubqn*$^{RNAi}$ X *w**/Y; *UAS-Tlk*$^{T2}$; *GMR-GAL4* | *UAS-Tlk*$^{T2}$/*UAS-ubqn*$^{RNAi}$; *GMR-GAL4/+* | *GMR>Tlk ubqn*$^{RNAi}$ |
| *w**; *UAS- Rm62*$^{RNAi}$ X *w**/Y; *UAS-Tlk*$^{T2}$; *GMR-GAL4* | *UAS-Tlk*$^{T2}$/*UAS- Rm62*$^{RNAi}$; *GMR-GAL4/+* | *GMR>Tlk Rm62*$^{RNAi}$ |
| *w*$^{1118}$ X *w*$^{*}$/Y; *eEF1α1*$^{LL06026}$ [f]/CyO *Kr>GFP* | *eEF1α1*$^{LL06026}$/+ | *eEF1α1*/+ |
| *w*$^{1118}$ X *w*$^{*}$/Y; *Df*(2R)ED2247 [f,g]/CyO *Kr>GFP* | Df(2)*eEF1α1*/+ | Df(2)*eEF1α1*/+ |
| *w*$^{1118}$ X *w**/Y; *P{w*$^{+mC}$*=GSV6}GS12243* [f]/*TM3 Sb*$^{1}$ *Kr>GFP* | *MEP-1*$^{GS12243}$/+ | *MEP-1*/+ |
| *w*$^{1118}$ X *w*$^{*}$/Y; *eEF1α1*$^{LL06026}$ *Hsc70-5*$^{LL00908}$ [f] /CyO *Kr>GFP* | *eEF1α1*$^{LL06026}$ *Hsc70-5*$^{LL00908}$/+ | *eEF1α1 Hsc70-5*/+ |
| *w*$^{1118}$ X *w*$^{*}$/Y; *Df*(2R)ED2247 *Hsc70-5*$^{LL00908}$/CyO *Kr>GFP* | *Df*(2)*eEF1α1 Hsc70-5*$^{LL00908}$/+ | *Df*(2)*eEF1α1 Hsc70-5*/+ |
| *w*$^{67c32}$ *Tlk*$^{\Delta14}$/*FM7 act-GFP* X *w*$^{*}$/Y; *eEF1α1*$^{LL06026}$/*CyO Kr>GFP* | *w*$^{67c32}$ *Tlk*$^{\Delta14}$/ *w*$^{*}$; *eEF1α1*$^{LL06026}$/+ | *Tlk*$^{\Delta14}$/+; *eEF1α1*/+ |
| *w*$^{67c32}$ *Tlk*$^{\Delta14}$/*FM7 act-GFP* X *w*$^{*}$/Y; *Df*(2R)ED2247 /*CyO Kr>GFP* | *w*$^{67c32}$ *Tlk*$^{\Delta14}$/ *w*$^{*}$; *Df*(2)*eEF1α1*/+ | *Tlk*$^{\Delta14}$/+; *Df*(2)*eEF1α1*/+ |
| *w*$^{67c32}$ *Tlk*$^{\Delta14}$/*FM7 act-GFP* X *w*$^{*}$/Y; *Hsc70-5*$^{LL00908}$/*CyO Kr>GFP* | *w*$^{67c32}$ *Tlk*$^{\Delta14}$/ *w*$^{*}$; *Hsc70-5*$^{LL00908}$/+ | *Tlk*$^{\Delta14}$/+; *Hsc70-5*/+ |
| *w*$^{1118}$ *Tlk*$^{27\text{–}9}$/*FM7 act-GFP* X *w*$^{*}$/Y; *eEF1α1*$^{LL06026}$/*CyO Kr>GFP* | *w*$^{1118}$ *Tlk*$^{27\text{–}9}$/ *w*$^{*}$; *eEF1α1*$^{LL06026}$/+ | *Tlk*$^{27\text{–}9}$/+; *eEF1α1*/+ |
| *w*$^{1118}$ *Tlk*$^{27\text{–}9}$/*FM7 act-GFP* X *w*$^{*}$/Y; *Df*(2R)ED2247/*CyO Kr>GFP* | *w*$^{1118}$ *Tlk*$^{27\text{–}9}$/ *w*$^{*}$; *Df*(2)*eEF1α1*/+ | *Tlk*$^{27\text{–}9}$/+; *Df*(2)*eEF1α1*/+ |

| | | |
|---|---|---|
| *w*$^{1118}$ *Tlk*$^{27–9}$/*FM7 act-GFP* X *w*$^{*}$/Y; *Hsc70-5*$^{LL00908}$/*CyO Kr>GFP* | *w*$^{1118}$ *Tlk*$^{27–9}$/ *w*$^{*}$; *Hsc70-5*$^{LL00908}$/+ | *Tlk*$^{27–9}$/+; *Hsc70-5*/+ |
| *w*$^{67c32}$ *Tlk*$^{\Delta14}$/*FM7 act-GFP* X *w*$^{*}$/Y; *eEF1α1*$^{LL06026}$ *Hsc70-5*$^{LL00908}$/*CyO Kr>GFP* | *w*$^{67c32}$ *Tlk*$^{\Delta14}$/ *w*$^{*}$; *eEF1α1*$^{LL06026}$ *Hsc70-5*$^{LL00908}$/+ | *Tlk*$^{\Delta14}$/+; *eEF1α1 Hsc70-5*/+ |
| *w*$^{67c32}$ *Tlk*$^{\Delta14}$/*FM7 act-GFP* X *w*$^{*}$/Y; *Df*(2R)ED2247 *Hsc70-5*$^{LL00908}$/*CyO Kr>GFP* | *w*$^{67c32}$ *Tlk*$^{\Delta14}$/ *w*$^{*}$; *Df*(2)*eEF1α1 Hsc70-5*$^{LL00908}$/+ | *Tlk*$^{\Delta14}$/+; *Df*(2)*eEF1α1 Hsc70-5*/+ |
| *w*$^{1118}$ *Tlk*$^{27–9}$/*FM7 act-GFP* X *w*$^{*}$/Y; *eEF1α1*$^{LL06026}$/*CyO Kr>GFP* | *w*$^{1118}$ *Tlk*$^{27–9}$/ *w*$^{*}$; *eEF1α1*$^{LL06026}$ *Hsc70-5*$^{LL00908}$/+ | *Tlk*$^{27–9}$/+; *eEF1α1 Hsc70-5*/+ |
| *w*$^{1118}$ *Tlk*$^{27–9}$/*FM7 act-GFP* X *w*$^{*}$/Y; *Df*(2R)ED2247 *Hsc70-5*$^{LL00908}$/*CyO Kr>GFP* | *w*$^{1118}$ *Tlk*$^{27–9}$/ *w*$^{*}$; *Df*(2)*eEF1α1 Hsc70-5*$^{LL00908}$/+ | *Tlk*$^{27–9}$/+; *Df*(2)*eEF1α1 Hsc70-5*/+ |
| *y*$^{1}$ *w**; *Tak1*$^{2}$ X *w*$^{*}$/Y; *eEF1α1*$^{LL06026}$/*CyO Kr>GFP* | *Tak1*$^{2}$/+; *eEF1α1*$^{LL06026}$/+ | *Tak1*$^{2}$/+; *eEF1α1*/+ |
| *y*$^{1}$ *w**; *Tak1*$^{2}$ X *w*$^{*}$/Y; *Df*(2R)ED2247/*CyO Kr>GFP* | *Tak1*$^{2}$/+; *Df*(2)*eEF1α1*/+ | *Tak1*$^{2}$/+; *Df*(2)*eEF1α1*/+ |
| *y*$^{1}$ *w**; *Tak1*$^{2}$ X *w*$^{*}$/Y; *Hsc70-5*$^{LL00908}$/*CyO Kr>GFP* | *Tak1*$^{2}$/+; *Hsc70-5*$^{LL00908}$/+ | *Tak1*$^{2}$/+; *Hsc70-5*/+ |
| *w**; *Tak1*$^{179}$ X *w*$^{*}$/Y; *eEF1α1*$^{LL06026}$/*CyO Kr>GFP* | *Tak1*$^{179}$/+; *eEF1α1*$^{LL06026}$/+ | *Tak1*$^{179}$/+; *eEF1α1*/+ |
| *w**; *Tak1*$^{179}$ X *w*$^{*}$/Y; *Df*(2R)ED2247/*CyO Kr>GFP* | *Tak179*/+; *Df*(2)*eEF1α1*/+ | *Tak1*$^{179}$/+; *Df*(2)*eEF1α1*/+ |
| *w**; *Tak1*$^{179}$ X *w*$^{*}$/Y; *Hsc70-5*$^{LL00908}$/*CyO Kr>GFP* | *Tak1*$^{179}$/+; *Hsc70-5*$^{LL00908}$/+ | *Tak1*$^{179}$/+; *Hsc70-5*/+ |
| *w*$^{67c32}$ *Tlk*$^{\Delta14}$/*FM7 act-GFP* X *w**/Y; *P{w*$^{+mC}$*=GSV6}GS12243/TM3 Sb*$^{1}$ *Kr>GFP* | *w*$^{67c32}$ *Tlk*$^{\Delta14}$/ *w**; *MEP-1*$^{GS12243}$/+ | *Tlk*$^{\Delta14}$/+; *MEP-1*/+ |
| *w*$^{1118}$ *Tlk*$^{27–9}$/*FM7 act-GFP* X *w**/Y; *P{w*$^{+mC}$*=GSV6}GS12243/TM3 Sb*$^{1}$ *Kr>GFP* | *w*$^{1118}$ *Tlk*$^{27–9}$/*w**; *MEP-1*$^{GS12243}$/+ | *Tlk*$^{27–9}$/+; *MEP-1*/+ |

[f]: To the lines requested from the stock centers, meiotic recombination was used to remove possible second site mutations.
[g]: *Df*(2R)ED2247 has a chromosomal aberration that approximately 37 genes are deleted including *eEF1α1*.

**A conceptual illustration of the dosage dependent genetic interaction experiment**

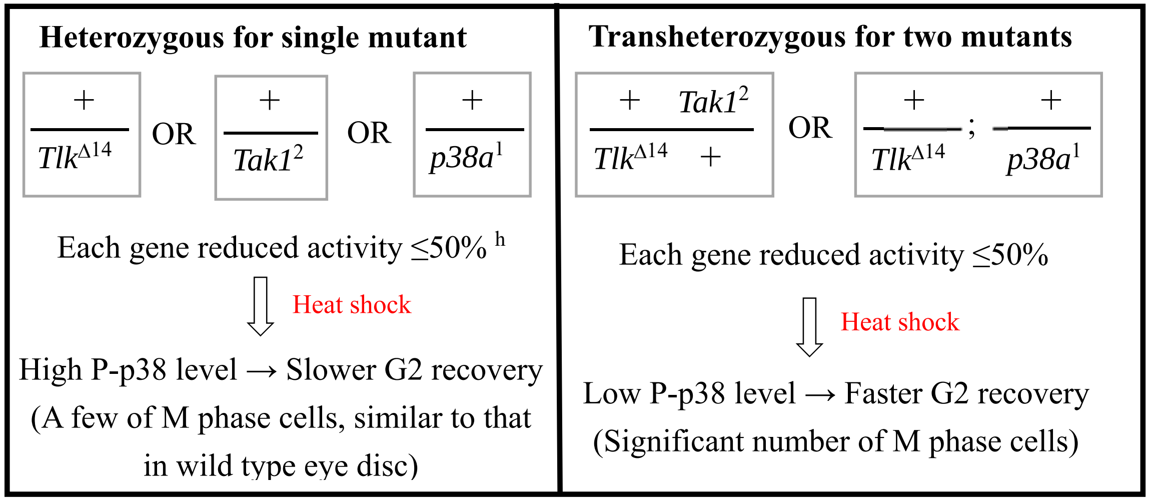


Indication:
The genetic interaction of *Tlk* with *Tak1* or *p38a* functions in the G2 phase.

[h]: Fly mutants, *p38a*$^{1}$, *p38a*$^{13}$, *Tlk*$^{\Delta 14}$ and *Tlk*$^{27\text{-}9}$, are amorphs (null alleles). The remaining activity from one copy of wild type gene is 50%. Mutants of other genes are hypomorphs, such as *eEF1α1*$^{LL06026}$, *Hsc70-5*$^{LL00908}$ and *Tak1*$^{2}$. Hypomorph describes a mutant in which the mutation in it does not completely deplete the gene activity in a homozygous fly. Therefore, the remaining activity from one copy of wild type gene is higher than 50% (>50%).

Table S2. Grouping by category was used to predict function of the 71 candidates

| Category | Term | Count | PValue | Genes | List Total | Pop Hits | Pop Total | Fold Enricl | Bonferroni | Benjamini | FDR |
|---|---|---|---|---|---|---|---|---|---|---|---|
| GOTERM_MF_FAT | GO:0000166~nucleotide binding | 30 | 1.39E-08 | Rm62, Act42A, CG2173, CG85258, eEf1α1, EF-4a, Hsp60, Hsp68, Hsp83, Hsc70-3, Hsc70-4, Hsc70-5, Aats-Ile, Mhc, Atpα, Cct5, Cctγ, Vha68-2, Brm, Myo10A, Zip | 66 | 1206 | 7918 | 2.984321 | 2.46E-06 | 3.07E-07 | 1.71E-05 |
| GOTERM_MF_FAT | GO:0051082~unfolded protein binding | 11 | 3.70E-10 | CG7033, CG8528, Gp93, Hsp60, Hsp68, Hsp83, Hsc70-4, Hsc70-5, Cct5, Cctγ, Tcp-1ζ | 66 | 75 | 7918 | 17.59556 | 6.55E-08 | 3.27E-08 | 4.54E-07 |
| GOTERM_BP_FAT | GO:0007052~mitotic spindle organization | 9 | 1.69E-04 | CG7033, CG8528, EF-4a, Hsp83, Cct5, Cctγ, Tcp-1ζ, EF4G, Sta | 66 | 194 | 7937 | 5.57896 | 0.10463433 | 0.0272525 | 0.25188494 |
| GOTERM_MF_FAT | GO:0008092~cytoskeletal protein binding | 7 | 0.0086861 | Mhc, Rfabg, Tm1, Tm2, α-Spec, α-Act, Zip | 66 | 218 | 7918 | 3.852238 | 0.78650977 | 0.0708932 | 10.1615551 |
| GOTERM_CC_FAT | GO:0044449~contractile fiber part | 5 | 5.24E-05 | Mhc, Prm, Tm1, Tm2, Zip | 59 | 18 | 4786 | 22.53296 | 0.00735793 | 0.0010545 | 0.06185745 |
| SP_PIR_KEYWORDS | coiled coil | 7 | 0.0012116 | eIF3-S9, MED23, Mhc, Prm, Tm1, Tm2, Zip | 73 | 215 | 12980 | 5.789105 | 0.14477212 | 0.0070833 | 1.39995488 |
| GOTERM_MF_FAT | GO:0008135~translation factor activity, nucleic acid binding | 6 | 5.66E-04 | EF1γ, eEF1α1, eIF3-S9, Trip1, EF4G | 66 | 83 | 7918 | 8.672508 | 0.0952849 | 0.0076731 | 0.69247882 |
| GOTERM_BP_FAT | GO:0009266~response to temperature stimulus | 7 | 2.64E-05 | Hsp60, Hsp68, Hsp83, Hsc70-3, Hsc70-5, Atpα, RpL40 | 66 | 73 | 7937 | 11.53155 | 0.01716954 | 0.0057563 | 0.03951163 |
| GOTERM_MF_FAT | GO:0003723~RNA bindin | 11 | 0.0259294 | Rm62, CG9143, EF-4a, EF3-S9, Fib, Pep, RpS3, EF4G, Mod, pAbp, Smn | 66 | 610 | 7918 | 2.163388 | 0.99043899 | 0.1582098 | 27.580223 |
| GOTERM_CC_FAT | GO:0035060~brahma com | 4 | 1.35E-04 | Bap60, Osa, Brm, Moi | 59 | 9 | 4786 | 36.05273 | 0.01882374 | 0.0018985 | 0.15909198 |
| GOTERM_BP_FAT | GO:0006325~chromatin organization | 7 | 0.0025676 | BEAF-32, Nurf-38, CG1716, RpL40, Osa, Brm, Moi | 66 | 170 | 7937 | 4.951783 | 0.81436463 | 0.1549818 | 3.76976153 |
| GOTERM_MF_FAT | GO:0004386~helicase acti | 5 | 0.0120909 | Rm62, CG2173, CG9143, EF-4a, | 66 | 109 | 7918 | 5.503197 | 0.88388091 | 0.0858075 | 13.8790044 |
| GOTERM_BP_FAT | GO:0010608~posttranscriptional regulation of gene expression | 5 | 0.0194716 | Rm62, EF3-S9, Hsc70-3, Hsc70-5, pAbp | 66 | 126 | 7937 | 4.772126 | 0.99999745 | 0.3490501 | 25.4650125 |
| Ungrouped genes (P>0.05) | | | | Prc, Gapdh2, Mor, CG10591, Rpn3, Rpn6, Fax, Rs1, MED14, Nop5, Cpr64Ad, EF1a, MEP-1, CG10576, Nopp140, Thiolase, CG7927, CG3018, CG14110, Actn, Set2, Glt, Muc68E | | | | | | | |

In each immunoprecipitation experiment, proteins with protein score >80 were selected. Proteins indentified by 4 times are marked in red.
To pedict function of the 71 candidates, ontology annotation was performed using the progam in DAVID bioinformatics resources (http://david.abcc.ncifcrf.gov/).
Proteins in each category were then modified according to information of Gene Ontology in Flybase.
